# Characterization, dissolution and solubility of the hydroxypyromorphite–hydroxyapatite solid solution [(Pb_x_Ca_1−x_)_5_(PO_4_)_3_OH] at 25 °C and pH 2–9

**DOI:** 10.1186/s12932-016-0034-8

**Published:** 2016-05-06

**Authors:** Yinian Zhu, Bin Huang, Zongqiang Zhu, Huili Liu, Yanhua Huang, Xin Zhao, Meina Liang

**Affiliations:** College of Environmental Science and Engineering, Guilin University of Technology, Guilin, 541004 People’s Republic of China; College of Light Industry and Food Engineering, Guangxi University, Nanning, 530004 People’s Republic of China

**Keywords:** Hydroxypyromorphite, Calcium hydroxyapatite, Solid solution, Dissolution, Lippmann diagram

## Abstract

**Background:**

The interaction between Ca-HAP and Pb^2+^ solution can result in the formation of a hydroxyapatite–hydroxypyromorphite solid solution [(Pb_x_Ca_1−x_)_5_(PO_4_)_3_(OH)], which can greatly affect the transport and distribution of toxic Pb in water, rock and soil. Therefore, it’s necessary to know the physicochemical properties of (Pb_x_Ca_1−x_)_5_(PO_4_)_3_(OH), predominantly its thermodynamic solubility and stability in aqueous solution. Nevertheless, no experiment on the dissolution and related thermodynamic data has been reported.

**Results:**

Dissolution of the hydroxypyromorphite–hydroxyapatite solid solution [(Pb_x_Ca_1−x_)_5_(PO_4_)_3_(OH)] in aqueous solution at 25 °C was experimentally studied. The aqueous concentrations were greatly affected by the Pb/(Pb + Ca) molar ratios (X_Pb_) of the solids. For the solids with high X_Pb_ [(Pb_0.89_Ca_0.11_)_5_(PO_4_)_3_OH], the aqueous Pb^2+^ concentrations increased rapidly with time and reached a peak value after 240–720 h dissolution, and then decreased gradually and reached a stable state after 5040 h dissolution. For the solids with low X_Pb_ (0.00–0.80), the aqueous Pb^2+^ concentrations increased quickly with time and reached a peak value after 1–12 h dissolution, and then decreased gradually and attained a stable state after 720–2160 h dissolution.

**Conclusions:**

The dissolution process of the solids with high X_Pb_ (0.89–1.00) was different from that of the solids with low X_Pb_ (0.00–0.80). The average *K*_sp_ values were estimated to be 10^−80.77±0.20^ (10^−80.57^–10^−80.96^) for hydroxypyromorphite [Pb_5_(PO_4_)_3_OH] and 10^−58.38±0.07^ (10^−58.31^–10^−58.46^) for calcium hydroxyapatite [Ca_5_(PO_4_)_3_OH]. The Gibbs free energies of formation (Δ*G*_*f*_^*o*^) were determined to be −3796.71 and −6314.63 kJ/mol, respectively. The solubility decreased with the increasing Pb/(Pb + Ca) molar ratios (X_Pb_) of (Pb_x_Ca_1‒x_)_5_(PO_4_)_3_(OH). For the dissolution at 25 °C with an initial pH of 2.00, the experimental data plotted on the Lippmann diagram showed that the solid solution (Pb_x_Ca_1−x_)_5_(PO_4_)_3_(OH) dissolved stoichiometrically at the early stage of dissolution and moved gradually up to the Lippmann *solutus* curve and the saturation curve for Pb_5_(PO_4_)_3_OH, and then the data points moved along the Lippmann *solutus* curve from right to left. The Pb-rich (Pb_x_Ca_1−x_)_5_(PO_4_)_3_(OH) was in equilibrium with the Ca-rich aqueous solution.

**Electronic supplementary material:**

The online version of this article (doi:10.1186/s12932-016-0034-8) contains supplementary material, which is available to authorized users.

**Graphical abstract:**
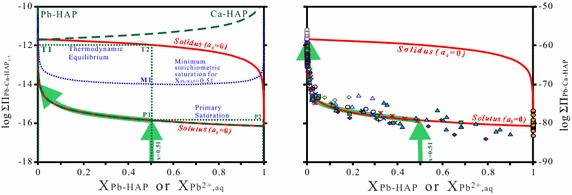
Lippmann diagrams for dissolution of the hydroxypyromorphite–hydroxyapatite solid solution [(Pb_x_Ca_1−x_)_5_(PO_4_)_3_OH] at 25 ˚C and an initial pH of 2.00.

## Background

The apatite group minerals with the general formula M_5_(PO_4_)_3_X have a wide compositional variation because of their huge isomorphic capacity and numerous substitutions of ions [1–5], which play an important role in many research areas, such as geology, environmental sciences, biomaterials, material science and technology [6–9].

Calcium hydroxyapatite [Ca-HAP] is the main component of vertebral animals’ bones [10–15]. Commonly, natural apatites as raw materials for the phosphate fertilizer industry contain some traces amount of various elements [10], among which lead and cadmium are predominantly risky and may be redistributed in natural waters, soil and agricultural products, especially in rice and vegetables. When these toxic heavy metals are taken into animals through food chains, they may concentrate in animals’ hard tissues through the possible substitution, which can cause osteoporotic processes and dental caries [10–13, 15].

Due to the large substitution capacity for various toxic trace elements, the natural or synthetic calcium apatite can be used to immobilize or remove hazardous chemicals in metal-contaminated soils and industrial wastewaters [4, 8, 11, 16–18]. Lead apatite is the most stable lead form under various environmental conditions. It is now considered that the in situ immobilization of lead-contaminated systems with phosphates is one of the appropriate and cost-effective technologies [19]. Two main mechanisms have been proposed for the immobilization of lead by hydroxyapatite, i.e., (1) hydroxyapatite dissolution, followed by phosphate reaction with dissolved Pb^2+^ and precipitation of pure hydroxypyromorphite [19, 20]; (2) ion exchange between Ca^2+^ ions in hydroxyapatite lattice and Pb^2+^ ions in solution [19, 21]. During the reaction of hydroxyapatite with Pb^2+^ solution, a new hydroxyapatite–hydroxypyromorphite solid solution [(Pb_x_Ca_1−x_)_5_(PO_4_)_3_(OH), Pb–Ca-HAP] with Pb^2+^ ions occupying Ca^2+^ sites formed and transformed in hydroxypyromorphite with times [22]. The existence of Pb–Ca-HAP as an intermediate phase was confirmed by X-ray diffractometer and electron microscopy analysis [23].

Solid solutions play a very important role in environmental and geochemical sciences because a metal-bearing solid solution may form on the solid surface when the solid come into contact with a metal-containing solution. The thermodynamic properties of the solid solution—aqueous solution equilibrium can greatly influence the transport and distribution of the toxic metals in water, rock and soil [24, 25]. Therefore, it’s necessary to know the physicochemical properties of the (Pb_x_Ca_1−x_)_5_(PO_4_)_3_(OH) solid solution, predominantly its thermodynamic solubility and stability in aqueous solution, whether for optimizing industrial processes relating to apatites, or for understanding mineral evolutions and natural phenomena [8]. Generally, the natural apatite is not a pure endmember but rather a solid solution [3]. Nevertheless, most of the researches about the apatite thermodynamic properties that have already been reported in literatures focus mainly on pure apatite [8, 16, 17, 26–29]. Until now, no experiment on the dissolution mechanism, solubility product and other thermodynamic data of the (Pb_x_Ca_1−x_)_5_(PO_4_)_3_(OH) solid solution [Pb–Ca-HAP] has been reported in literatures, even though the dissolution-related release of lead and phosphate from solid to solution has a potential effect on the cycling of the relevant elements.

In the present study, lead hydroxyapatite [hydroxypyromorphite, Pb-HAP, Pb_5_(PO_4_)_3_(OH)], lead–calcium hydroxyapatite solid solution [Pb–Ca-HAP (Pb_x_Ca_1−x_)_5_(PO_4_)_3_(OH)] with varying Pb/(Pb + Ca) molar ratios and calcium hydroxyapatite [Ca-HAP, Ca_5_(PO_4_)_3_(OH)] were firstly synthesized and characterized by chemical analysis, powder X-ray diffraction (XRD), Fourier-transform infrared spectroscopy (FT-IR), field emission scanning electron microscopy (FE-SEM) and field emission transmission electron microscopy (FE-TEM), and then the dissolution and release processes of elements (Pb^2+^, Ca^2+^, PO_4_^3−^) were investigated through batch experiments. The Lippmann diagram [30] for the (Pb_x_Ca_1−x_)_5_(PO_4_)_3_(OH) solid solution was constructed to study the reaction path of the solid-water interaction and its possible effect on the solubility and distribution of lead and phosphate in the environment.

## Experimental methods

### Solid preparation and characterization

#### Solid preparation

The Pb-HAP, Pb–Ca-HAP solid solution and Ca-HAP samples were synthesized according to the following precipitation reaction: 5M^2+^+3PO_4_^3−^+OH^−^ = M_5_(PO_4_)_3_OH, where M = (Pb + Ca) for the solid solution and Pb or Ca for the end-member. Firstly, a series of 250 mL solutions of different Pb/(Pb + Ca) molar ratios were prepared by dissolving different amounts of Pb(CH_3_COO)_2_·H_2_O and Ca(CH_3_COO)_2_·3H_2_O into pure water, while the total amount of lead and calcium in each solution was maintained to be 0.4 mol/L. Two hundred and fifty millilitre of 4.4 mol/L CH_3_COONH_4_ buffer solution was then mixed with each lead–calcium solution in 1L polypropylene bottle. After that, 500 mL of 0.12 mol/L NH_4_H_2_PO_4_ solution was quickly added into the bottle with stirring (Table 1). The resulting white suspension was adjusted to pH 7.50 with NH_4_OH, stirred for 10 min at room temperature, and then aged at 100 °C for 48 h, as suggested by Yasukawa et al. [10]. Finally, the obtained precipitates were carefully washed with pure water and dried in an oven at 70 °C for 16 h.

**Table 1:** Summary of synthesis and composition of the hydroxypyromorphite–hydroxyapatite solid solution [(Pb_x_Ca_1−x_)_5_(PO_4_)_3_OH]

Sample No.	Volumes of the precursors (mL)				Solid composition	Residual solid composition after dissolution at 25 °C and an initial pH of 2.00 for 300 days
	0.4 M	0.4 M	4.4 M	0.12 M		
	Pb(CH_3_COO)_2_·2H_2_O	Ca(CH_3_COO)_2_·H_2_O	CH_3_COONH_4_	NH_4_H_2_PO_4_		
Pb–Ca-HAP-00	0	250	250	500	(Pb_0.00_Ca_1.00_)_5_(PO_4_)_3_OH	(Pb_0.00_Ca_1.00_)_5_(PO_4_)_3_OH
Pb–Ca-HAP-01	25	225	250	500	(Pb_0.10_Ca_0.90_)_5_(PO_4_)_3_OH	(Pb_0.10_Ca_0.90_)_5_(PO_4_)_3_OH
Pb–Ca-HAP-02	50	200	250	500	(Pb_0.20_Ca_0.80_)_5_(PO_4_)_3_OH	(Pb_0.21_Ca_0.79_)_5_(PO_4_)_3_OH
Pb–Ca-HAP-03	75	175	250	500	(Pb_0.30_Ca_0.70_)_5_(PO_4_)_3_OH	(Pb_0.32_Ca_0.68_)_5_(PO_4_)_3_OH
Pb–Ca-HAP-04	100	150	250	500	(Pb_0.41_Ca_0.59_)_5_(PO_4_)_3_OH	(Pb_0.44_Ca_0.56_)_5_(PO_4_)_3_OH
Pb–Ca-HAP-05	125	125	250	500	(Pb_0.51_Ca_0.49_)_5_(PO_4_)_3_OH	(Pb_0.54_Ca_0.46_)_5_(PO_4_)_3_OH
Pb–Ca-HAP-06	150	100	250	500	(Pb_0.61_Ca_0.39_)_5_(PO_4_)_3_OH	(Pb_0.66_Ca_0.34_)_5_(PO_4_)_3_OH
Pb–Ca-HAP-07	175	75	250	500	(Pb_0.69_Ca_0.31_)_5_(PO_4_)_3_OH	(Pb_0.77_Ca_0.23_)_5_(PO_4_)_3_OH
Pb–Ca-HAP-08	200	50	250	500	(Pb_0.80_Ca_0.20_)_5_(PO_4_)_3_OH	(Pb_0.88_Ca_0.12_)_5_(PO_4_)_3_OH
Pb–Ca-HAP-09	225	25	250	500	(Pb_0.89_Ca_0.11_)_5_(PO_4_)_3_OH	(Pb_0.95_Ca_0.05_)_5_(PO_4_)_3_OH
Pb–Ca-HAP-10	250	0	250	500	(Pb_1.00_Ca_0.00_)_5_(PO_4_)_3_OH	(Pb_1.00_Ca_0.00_)_5_(PO_4_)_3_OH

#### Characterization

To determine the chemical component of each obtained precipitate, 10 mg of the precipitate was firstly dissolved in 20 mL of 1 mol/L nitric acid solution and diluted to 100 mL with pure water. The Pb^2+^, Ca^2+^and PO_4_^3−^ concentrations were then measured by the inductively coupled plasma—optical emission spectrometer (ICP-OES, Perkin-Elmer Optima 7000DV). All solid samples were also characterized using an X’Pert PRO powder X-ray diffractometer (XRD) with Cu Kα radiation (40 kV and 40 mA) at a scanning rate of 0.10°/min in a 2θ range of 10–80°. By comparing the recorded XRD pattern with the standard from the International Center for Diffraction Data (ICDD), the precipitates were crystallographically identified. Using the Fourier transform infrared spectrophotometer (FT-IR, Nicolet Nexus 470), all solids were also analyzed in KBr pellets within 4000–400 cm^−1^. The field-emission scanning electron microscope (FE-SEM, Hitachi S-4800) and the field-emission transmission electron microscope (FE-TEM, Jeol JEM-2100F) were applied to observe the solid morphology.

### Dissolution experiments

2.0 g of each Ca-HAP, Pb–Ca-HAP or Pb-HAP solid was first added into a series of 100 mL polypropylene bottles, which were then filled with 100 mL of HNO_3_ solution (pH 2.00), ultrapure water (pH 5.60) or NaOH solution (pH 9.00). All bottles were capped and placed in water baths at 25 °C. From each bottle, the aqueous solutions (5 mL) were sampled at 22 time intervals (1, 3, 6, 12, 24, 48, 72, 120, 240, 360, 480, 720, 1080, 1440, 1800, 2160, 2880, 3600, 4320, 5040, 5760, 7200 h), filtered through 0.22 μm pore filters and stabilized in 25 mL volumetric flask using 0.2 % HNO_3_. An equivalent volume of pure water (5 mL) was added into the bottle after each sampling. The dilution effects of the acidic and basic solutions throughout the experiments were considered in the calculation by using the program PHREEQC [31]. The aqueous concentrations of Pb, Ca and P were measured using ICP-OES. At the end of the dissolution experiment, the solids were collected from the bottles, rinsed, dried and characterized using XRD, FT-IR, FE-SEM and FE-TEM instruments in the same manner as previously described.

### Thermodynamic calculations

The aqueous activities of Pb^2+^(aq), Ca^2+^(aq), PO_4_^3−^(aq), and OH^−^(aq) were first calculated using PHREEQC Version 3 [31], and then the ion activity products (IAPs) for (Pb_x_Ca_1−x_)_5_(PO_4_)_3_(OH) were calculated according to the mass-action expressions. The minteq.v4.dat database with the addition of the thermodynamic data for PbHPO_4_^0^, PbH_2_PO_4_^+^ and PbP_2_O_7_^2−^ from the llnl.dat database was used in the simulation. The minteq.v4.dat database contains thermodynamic data for the aqueous species and gas and mineral phases that are derived from the database files of MINTEQA2 [32, 33]. The aqueous species considered in the calculation included Pb^2+^, PbOH^+^, Pb(OH)_2_^0^, Pb(OH)_3_^−^, Pb(OH)_4_^2−^, Pb_3_(OH)_4_^2+^, Pb_2_OH^3+^, Pb_4_(OH)_4_^4+^, PbHPO_4_^0^, PbH_2_PO_4_^+^ and PbP_2_O_7_^2−^ for the total lead; Ca^2+^, CaOH^+^, CaHPO_4_, CaPO_4_^−^ and CaH_2_PO_4_^+^ for the total calcium calculation. For the total phosphate, the aqueous species considered were PO_4_^3−^, HPO_4_^2−^, H_2_PO_4_^−^, H_3_PO_4_^0^, PbHPO_4_^0^, PbH_2_PO_4_^+^, PbP_2_O_7_^2−^, CaHPO_4_, CaPO_4_^−^ and CaH_2_PO_4_^+^.

## Results and discussion

### Solid characterization

The chemical compositions of the prepared solids are related to the Pb/(Pb + Ca) molar ratios in the precursor solutions (Table 1). The compositions of the Pb-HAP, Pb–Ca-HAP and Ca-HAP precipitates obtained are the designed components of (Pb_x_Ca_1−x_)_5_(PO_4_)_3_(OH) with the (Pb + Ca)/P molar ratio of 1.67, and all of the Pb/(Pb + Ca) molar ratios are almost the same as the precursor solutions.

**Fig. 1:**
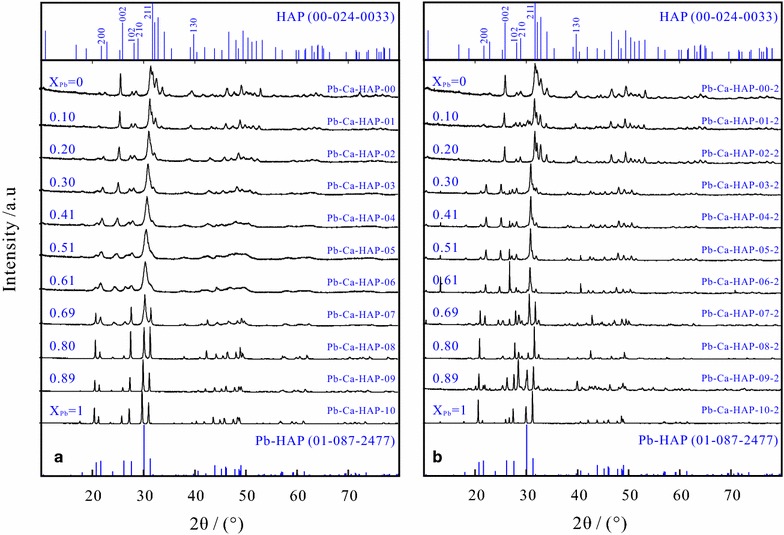
X-ray diffractograms (XRD) of the hydroxypyromorphite–hydroxyapatite solid solution [(Pb_x_Ca_1−x_)_5_(PO_4_)_3_OH] before (**a**) and after dissolution at 25 °C and an initial pH of 2.00 for 300 days (**b**)

The XRD patterns showed that all (Pb_x_Ca_1−x_)_5_(PO_4_)_3_(OH) solids belong to the apatite group of the hexagonal system P6_3_/m differing only in peak location, peak width and absolute intensity (Fig. 1). The solid with X_Pb_ = 1.00 is identified as lead phosphate hydroxide [hydroxypyromorphite, Pb-HAP] (Reference code 01-087-2477) with the calculated unit cell parameters of *a* = 0.989 nm and *c* = 0.748 nm, and the solid with X_Pb_ = 0.00 is recognized as calcium phosphate hydroxide [calcium hydroxyapatite, Ca-HAP] (Reference code 00-024-0033) with the calculated unit cell parameters of *a* = 0.944 nm and *c* = 0. 0.686 nm. Due to the substitution of Ca^2+^ (0.100 nm) with larger Pb^2+^ (0.119 nm) in the apatite structure [2, 10, 13], the lattice parameters *a* and *c* increased almost linearly with the increasing X_Pb_ from 0.944 to 0.989 nm and from 0.686 to 0.748 nm, respectively. However, an obvious deviation of both *a* and *c* lattice parameters from Vegard’s rule was also observed [34]. The reflection of the (Pb_x_Ca_1−x_)_5_(PO_4_)_3_(OH) solid shifts gradually to a higher-angle direction as the solid Pb/(Pb + Ca) molar ration (X_Pb_) decreases, which indicated that (Pb_x_Ca_1−x_)_5_(PO_4_)_3_(OH) is a continuous solid solution within the whole range of X_Pb_ = 0–1.00 (Fig. 1). Some additional peaks other than hydroxypyromorphite have been also recognized in XRD patterns after the dissolution at initial pH 2.00 and 25 °C (Fig. 1), the peaks of PbHPO_4_ [lead hydrogen phosphate, Reference code 00-029-0773] around 13.155° [2θ], the peaks of Pb_3_(PO_4_)_2_ [lead phosphate, Reference code 00-025-1394] around 26.783, 28.559 and 29.250° [2θ] were also recognized, which means that PbHPO_4_ and Pb_3_(PO_4_)_2_ as secondary precipitate formed during the (Pb_x_Ca_1−x_)_5_(PO_4_)_3_(OH) dissolution at the initial pH 2.00. But no secondary minerals were observed after the dissolution at the initial pHs 5.60 and 9.00 (Additional file 1: Appendix A). The result of the PHREEQC simulation also shows that the aqueous solutions were undersaturated with respect to any possible secondary minerals (e.g., massicot (PbO), litharge (PbO), PbO·0.3H_2_O, plattnerite (PbO_2_), Pb(OH)_2_, Pb_2_O(OH)_2_, PbHPO_4_, Pb_3_(PO_4_)_2_; lime [CaO], portlandite [Ca(OH)_2_], Ca_3_(PO_4_)_2_(beta), CaHPO_4_, CaHPO_4_·2H_2_O, Ca_4_H(PO_4_)_3_·3H_2_O), except in some cases of the dissolution at the initial pH 2.00, in which the aqueous solutions were saturated or nearly saturated with respect to PbHPO_4_ and Pb_3_(PO_4_)_2_.

**Fig. 2:**
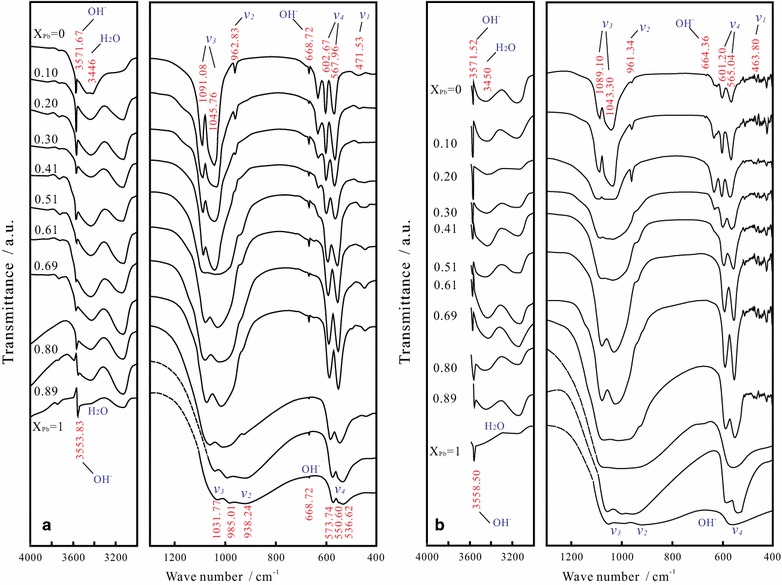
Fourier transform infrared (FT-IR) spectra of the hydroxypyromorphite–hydroxyapatite solid solution [(Pb_x_Ca_1−x_)_5_(PO_4_)_3_OH] before (**a**) and after dissolution at 25 °C and an initial pH of 2.00 for 300 days (**b**)

Although lead hydroxyapatite (Pb-HAP) and calcium hydroxyapatite (Ca-HAP) are isomorphous, their FT-IR spectra have essential differences. Generally, the tetrahedral PO_4_^3−^ has four vibrational modes, i.e., the symmetric P–O stretching (*v*_1_), the O–P–O bending (*v*_2_), the P–O stretching (*v*_3_), and the O–P–O bending (*v*_4_). But in the undistorted state, only the absorptions for the vibrations *v*_3_ and *v*_4_ can be detected, the two other vibrations *v*_1_ and *v*_2_ become infrared inactive [12]. In the FT-IR spectra, the tetrahedral PO_4_^3−^ of Ca-HAP showed the vibrational bands at 962.83 cm^−1^ (*ν*_2_), 1045.76 and 1091.08 cm^−1^ (*ν*_3_), 567.96 and 602.67 cm^−1^ (*ν*_4_), which shifted to 938.24 cm^−1^ (*ν*_2_), 985.01 and 1031.77 cm^−1^ (*ν*_3_), 536.62–573.74 cm^−1^ (*ν*_4_) as the solid Pb/(Pb + Ca) molar ratio (X_Pb_) increased from 0 to 1.00, respectively (Fig. 2). The bands at 471.53 cm^−1^ (*ν*_1_) and 633.05 cm^−1^ (*ν*_4_) diminish with increasing X_Pb_ and disappear as X_Pb_ > 0.80 because of the variation of the PO_4_^3−^ symmetry. All bands, especially the P–O stretching (*v*_3_) bands, weaken with the increasing X_Pb_ due to the IR beam scattering of large particles [10]. The strong sharp bands at 3553.83–3571.67 cm^−1^ represent the stretching vibrations of the bulk OH^−^ and the band at 3735.15–3736.56 cm^−1^ represents the surface P-OH groups [15, 35]. The band at 1455 cm^−1^ for CO_3_^2−^ vibration [36] and the band at 871 cm^−1^ for HPO_4_^2−^ [10, 11] are not visible in the FT-IR spectra of the present work.

**Fig. 3:**
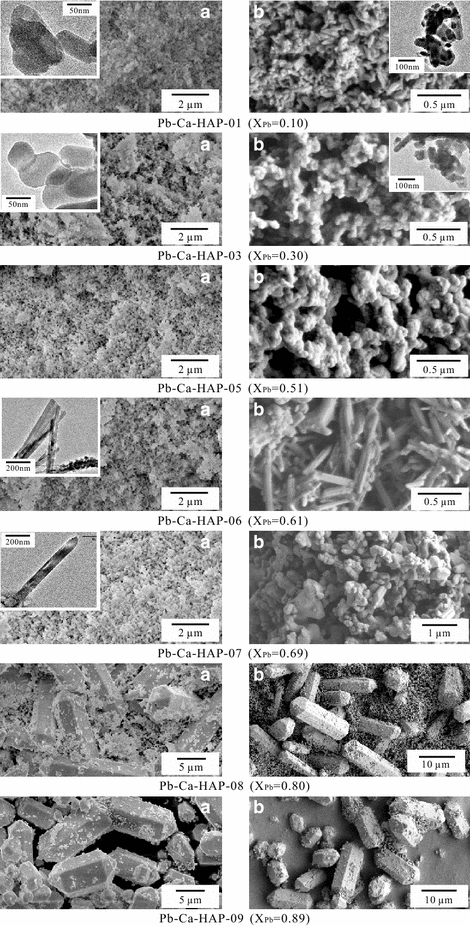
Field emission scanning electron micrographs (FE-SEM) and transmission electron microscope (TEM) images‎ of the hydroxypyromorphite–hydroxyapatite solid solution [(Pb_x_Ca_1−x_)_5_(PO_4_)_3_OH] before (**a**) and after dissolution at 25 °C and an initial pH of 2.00 for 300 days (**b**)

The Pb/(Pb + Ca) atomic ratio (X_Pb_) can greatly affect the morphology and crystal structure of the (Pb_x_Ca_1−x_)_5_(PO_4_)_3_(OH) solid solution [2, 10, 13, 37]. With the increasing X_Pb_, the lattice parameters increased gradually accompanying morphology variation (Fig. 3). The (Pb_x_Ca_1−x_)_5_(PO_4_)_3_(OH) solids with X_Pb_ = 0–0.51 are usually prism crystals with a hexagonal pyramid as termination (particle size 50–100 nm); the solids with X_Pb_ = 0.61–0.69 are typically hexagonal columnar crystals with a hexagonal pyramid or a pinacoid as termination, which elongate along the *c* axis (200–600 nm); the solids with X_Pb_ = 0.80–1.00 are characteristically prism crystals with a hexagonal pyramid as termination (2–20 µm) [34]. The hydroxypyromorphite (Pb-HAP) particles have an average length and width of 7.00 µm (3.43–10.43 µm) and 3.76 µm (1.83–4.88) before dissolution, and 6.85 µm (3.81–11.87 µm) and 4.04 µm (2.96–5.14 µm) after dissolution at 25 °C and an initial pH of 2.00.

### Dissolution mechanism

**Fig. 4:**
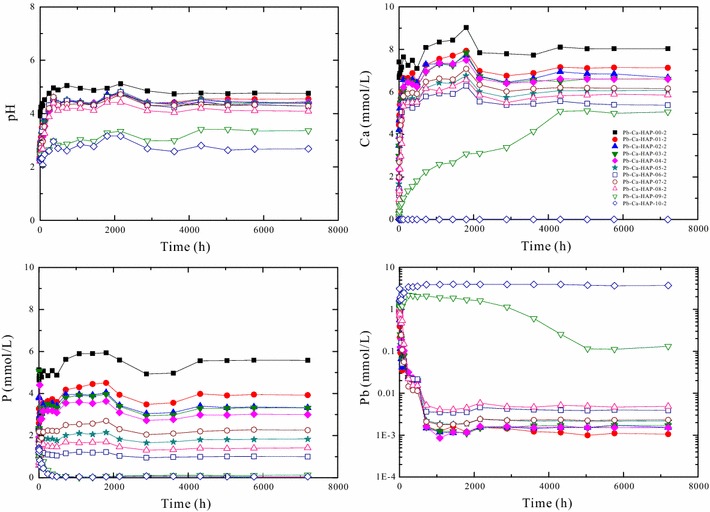
Change of the solution pH and elemental concentrations with time for dissolution of the hydroxypyromorphite–hydroxyapatite solid solution [(Pb_x_Ca_1−x_)_5_(PO_4_)_3_OH] at 25 °C and an initial pH of 2.00 for 300 days

**Fig. 5:**
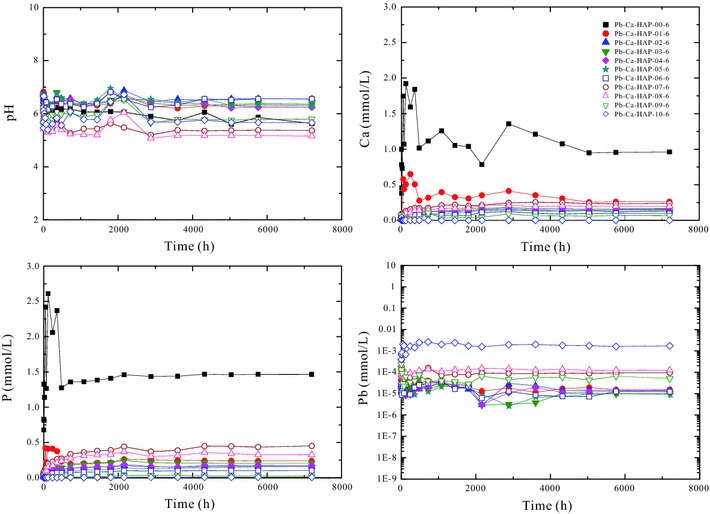
Change of the solution pH and elemental concentrations with time for dissolution of the hydroxypyromorphite–hydroxyapatite solid solution [(Pb_x_Ca_1−x_)_5_(PO_4_)_3_OH] at 25 °C and an initial pH 5.60 for 300 days

**Fig. 6:**
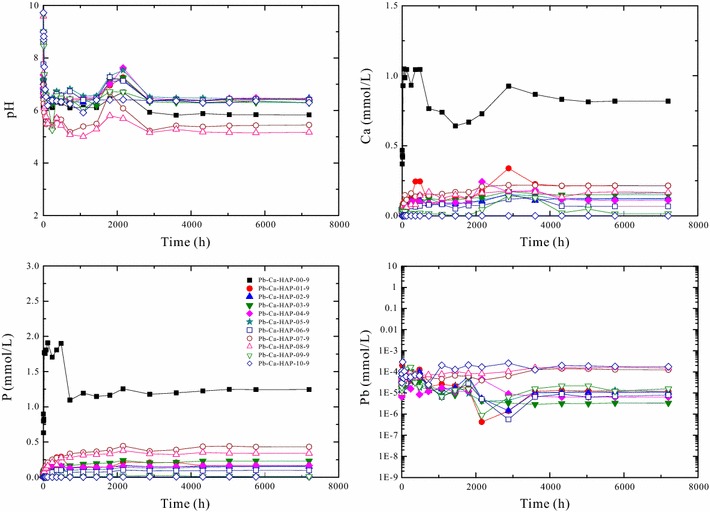
Change of the solution pH and elemental concentrations with time for dissolution of the hydroxypyromorphite–hydroxyapatite solid solution [(Pb_x_Ca_1−x_)_5_(PO_4_)_3_OH] at 25 °C and an initial pH of 9.00 for 300 days

The solution pH and aqueous element concentrations for the dissolution of (Pb_x_Ca_1−x_)_5_(PO_4_)_3_(OH) [Pb–Ca-HAP] at 25 °C and different initial pHs (2.00, 5.60 and 9.00) versus time are illustrated in Figs. 4, 5 and 6.

Dissolution of (Pb_x_Ca_1−x_)_5_(PO_4_)_3_(OH) in the acidic solution is stoichiometric in the early stage of dissolution and then always non-stoichiometric to the end of the dissolution experiments. For the dissolution at 25 °C and an initial pH of 2.00 (Fig. 4), the solution pH increased from 2.00 to 2.96–4.96 after 360 h dissolution and reached a stable state with pH 2.63–4.77 after 5040 h dissolution. The Pb/(Pb + Ca) atomic ratios (X_Pb_) of the (Pb_x_Ca_1−x_)_5_(PO_4_)_3_(OH) solids can greatly affect the element concentrations in the aqueous solutions. In general, the final solution pHs decrease with the increasing X_Pb_ of the solids.

The dissolution process of the (Pb_x_Ca_1−x_)_5_(PO_4_)_3_(OH) solids with high X_Pb_ (0.89–1.00) is different from that of the (Pb_x_Ca_1−x_)_5_(PO_4_)_3_(OH) solids with low X_Pb_ (0.00–0.80) (Fig. 4). For the solids with high X_Pb_ or low X_Ca_ [(Pb_0.89_Ca_0.11_)_5_(PO_4_)_3_OH], the aqueous Ca^2+^ concentrations increased gradually with the dissolution time and achieved a stable state after 4320 h dissolution. The aqueous Pb^2+^ concentrations increased rapidly with the dissolution time and achieved a peak value within 240–720 h, and then decreased gradually and attained a stable state after 5040 h dissolution. The aqueous phosphate concentration increased rapidly with time and achieved a peak value within 1–12 h, and then decreased gradually and attained a stable state after 2160 h dissolution. For the hydroxypyromorphite dissolution at 25 °C and an initial pH of 2.00 (Fig. 4), the aqueous lead concentrations increased constantly and reached a stable state after 720 h dissolution; the phosphate could be quickly released and reached the peak solution concentrations within 1 h dissolution, and then the aqueous phosphate concentration decreased and reached a stable state after 720 h; the solution pHs increased from 2.00 to 2.96 within 360 h and then varied between 2.58 and 3.16 (Fig. 4).

For the (Pb_x_Ca_1−x_)_5_(PO_4_)_3_(OH) solids with low X_Pb_ (0.00–0.80) or high X_Ca_, the aqueous Ca^2+^ concentrations increased slowly with time and reached a peak value after 1200–1800 h dissolution, and then decreased slightly and were relatively stable after 4320 h. The aqueous Pb^2+^ concentrations increased quickly with time and reached a peak value within 1–12 h, and then decreased gradually and attained a stable state after 720–2160 h dissolution. The aqueous phosphate concentrations showed a similar evolution trend to that of the aqueous Ca^2+^ concentrations.

At the early stage of the dissolution (within 1 h), the aqueous Pb/(Pb + Ca) molar ratios (X_Pb,aq_) are almost equal to the stoichiometric Pb/(Pb + Ca) atomic ratios (X_Pb,aq_) of the corresponding (Pb_x_Ca_1−x_)_5_(PO_4_)_3_(OH) solids. Then, the aqueous Pb/(Pb + Ca) molar ratios (X_Pb,aq_) decreased with time and were lower than the stoichiometric Pb/(Pb + Ca) ratios of the corresponding solids (X_Pb_) (Additional file 2: Appendix B). For the solids with high X_Pb_ or low X_Ca_ [(Pb_0.89_Ca_0.11_)_5_(PO_4_)_3_OH], the aqueous Pb/(Pb + Ca) molar ratios (X_Pb,aq_) decreased gradually from 0.90 to 0.02 with the increasing time and achieved a stable state after 5040 h dissolution. For the solids with low X_Pb_ (0.00–0.80), the aqueous Pb/(Pb + Ca) molar ratios (X_Pb,aq_) decreased rapidly from 0.00–0.79 to 0.00–0.004 after 72 h dissolution and then achieved a stable state.

The difference in the dissolution processes between the solids with X_Pb_ of 0.00–0.80 and those with X_Pb_ of 0.89–1.00 is related to the differences in the crystal structure and morphology of the (Pb_x_Ca_1−x_)_5_(PO_4_)_3_(OH) solids (Figs. 1, 2, 3). Crystallographically, two independent metal atoms, i.e., the M(1) atom and the M(2) atom, exist in the HAP lattice. Six O atoms and an OH surrounded the M(2) atom, while only six O atoms surrounded the M(1) atom almost octahedrally. Larger Pb^2+^ cations prefer to occupy the M(2) sites and smaller Ca^2+^ cations prefer to occupy the M(1) sites in the apatite structure. When Pb^2+^ cations substitute for Ca^2+^ cations in the apatite lattice, they occupied almost solely the M(2) sites, until, at X_Pb_ > 0.4, they also began to occupy the M(1) sites considerably, which could explain the discontinuity at around X_Pb_ = 0.4–0.6 in the curves of the *a* and *c*-axis parameters versus X_Pb_ [34]. The greatest deviations were noted at an intermediate X_Pb_, whereas the entire replacement by Pb^2+^ formed a crystal that had the apatite structure, despite a total enlargement of the unit cell because of the larger Pb^2+^ cations [11, 16, 17, 27–29, 38], or the change of the *a*-axis parameter had a break at X_Pb_ of 0.8 [11]. For the dissolution of the (Pb_x_Ca_1−x_)_5_(PO_4_)_3_(OH) solids with high X_Pb_ in the acidic solution, Pb^2+^ cations, which occupy nearly all the M(2) sites [2, 39], can be preferentially released because of the interaction of the solution H^+^ with the OH surrounding the M(2) atom. For the dissolution of the (Pb_x_Ca_1−x_)_5_(PO_4_)_3_(OH) solids with low X_Pb_ in the acidic solution, Ca^2+^ cations in the M(2) sites can be preferentially released with respect to Pb^2+^ cations in the M(2) sites [2, 39], which will cause a higher aqueous X_Ca,aq_ than the solid X_Ca_ during the initial period of dissolution.

For the (Pb_x_Ca_1−x_)_5_(PO_4_)_3_(OH) dissolution in pure water (pH 5.60) and the solution of initial pH 9.00, the solution pH values, lead and phosphate concentrations reached a stable state after 5040 h dissolution, which indicated a possible attainment of a steady-state between the (Pb_x_Ca_1−x_)_5_(PO_4_)_3_(OH) solid and the aqueous solution (Figs. 5, 6). The solution lead and phosphate concentrations are smaller than those for the (Pb_x_Ca_1−x_)_5_(PO_4_)_3_(OH) dissolution in pure water at an initial pH of 2.00, the solubility of (Pb_x_Ca_1−x_)_5_(PO_4_)_3_OH [Pb–Ca-HAP] at an initial pH of 5.60 or 9.00 is significantly lower that that at an initial pH of 2.00 (Figs. 5, 6).

For the (Pb_x_Ca_1−x_)_5_(PO_4_)_3_(OH) dissolution at an initial pH of 2.00 or 5.60, all solution pHs are higher than the initial pH values. pH of final solutions is buffered by various species of phosphates. The significant H^+^ consuming at the beginning of the dissolution indicates that the H^+^ sorption onto negatively charged oxygen ions of phosphate groups of the solid solution (Pb_x_Ca_1−x_)_5_(PO_4_)_3_OH may result in the transforming of PO_4_^3−^ into HPO_4_^2−^ at the solid surface in the acidic solution and promote the dissolution process. Additionally, the depleting of H^+^ ions during the solid dissolution may also result from the coexisting exchange of 2H^+^ for Pb^2+^ and Ca^2+^ at the (Pb_x_Ca_1−x_)_5_(PO_4_)_3_(OH) surface. Therefore, a complete describing of the (Pb_x_Ca_1−x_)_5_(PO_4_)_3_(OH) dissolution should include following processes: (I) Diffusion of H^+^ from solution to the solid-solution interface; (II) H^+^ adsorption/desorption at the (Pb_x_Ca_1−x_)_5_(PO_4_)_3_(OH) surface; (III) Protonation and transformation of PO_4_^3−^ into HPO_4_^2−^ at the (Pb_x_Ca_1−x_)_5_(PO_4_)_3_(OH) surface in the acidic solution; (IV) Stoichiometric desorption of Pb^2+^, Ca^2+^ and PO_4_^3−^ from the (Pb_x_Ca_1−x_)_5_(PO_4_)_3_(OH) surface and complexation; (V) Re-adsorption of Pb^2+^ and/or PO_4_^3−^ from solution back onto the (Pb_x_Ca_1−x_)_5_(PO_4_)_3_(OH) surface; (VI) Attaining of a stable state.

In process (I)–(III), the diffusion and adsorption of protons onto the (Pb_x_Ca_1−x_)_5_(PO_4_)_3_(OH) surface can increase the solution pH from 2.00 to 2.96–4.96 within 360 h for the dissolution at an initial pH of 2.00. In process (IV) and (V), Pb^2+^, Ca^2+^ and PO_4_^3−^ can be released from the (Pb_x_Ca_1−x_)_5_(PO_4_)_3_(OH) surface to the aqueous solution. Many possible reactions should be considered in describing the apatite dissolution due to its structural complexity [7]. The reaction (1) for the (Pb_x_Ca_1−x_)_5_(PO_4_)_3_(OH) dissolution is strongly affected by the initial solution pH and the protonation and complexation reactions (2)–(6), which can result in an increase of the aqueous pH for the (Pb_x_Ca_1−x_)_5_(PO_4_)_3_(OH) dissolution in acidic solution or a decrease of the solution pH for the (Pb_x_Ca_1−x_)_5_(PO_4_)_3_(OH) dissolution in alkali solution.

1 $$\begin{aligned} &\left( {{\text{Pb}}_{\text{x}} {\text{Ca}}_{{ 1- {\text{x}}}} } \right)_{5} \left( {{\text{PO}}_{4} } \right)_{3} {\text{OH }} \\ & \quad = \, 5{\text{xPb}}^{2 + } + \, \left( {5 - 5{\text{x}}} \right){\text{Ca}}^{2 + } + \, 3{\text{PO}}_{4}^{3 - } + {\text{ OH}}^{ - }\end{aligned}$$

2 $${\text{PO}}_{4}^{3 - } + {\text{ nH}}^{ + } \leftrightarrow {\text{ H}}_{\text{n}} {\text{PO}}_{ 4}^{{\left( {3 - {\text{n}}} \right) - }} \quad \left( {{\text{n}} = 1, \, 2, \, 3} \right)$$

3 $${\text{Pb}}^{2 + } + {\text{nOH}}^{ - } \leftrightarrow {\text{ Pb}}\left( {\text{OH}} \right)_{\text{n}}^{{({\text{n}} - 2) - }} \quad \left( {{\text{n}}\;{ = }\;1,\;2,\;3,\;4} \right)$$

4 $${\text{Pb}}^{2 + } + {\text{ H}}_{\text{n}} {\text{PO}}_{ 4}^{{\left( {3 - n} \right) - }} \leftrightarrow {\text{ PbH}}_{\text{n}} {\text{PO}}_{ 4}^{{({\text{n}} - 1) + }} \quad \left( {{\text{n}}\;{ = }\;1,\;2} \right)$$

5 $${\text{Ca}}^{2 + } + \;{\text{OH}}^{ - } \leftrightarrow {\text{ Ca}}\left( {\text{OH}} \right)^{ + }$$

6 $${\text{Ca}}^{2 + } + {\text{ H}}_{\text{n}} {\text{PO}}_{4}^{{\left( {3 - {\text{n}}} \right) - }} \leftrightarrow {\text{ CaH}}_{\text{n}} {\text{PO}}_{4}^{{\left( {1 - {\text{n}}} \right) - }} \quad \left( {{\text{n}} = 0, \, 1, \, 2} \right)$$

In process (V), for (Pb_x_Ca_1−x_)_5_(PO_4_)_3_(OH) with X_Pb_ ≤ 0.80, Pb^2+^ ions are re-absorbed non-stoichiometrically from the solution onto the (Pb_x_Ca_1−x_)_5_(PO_4_)_3_(OH) surface, the aqueous lead concentrations and the solution Pb/(Pb + Ca) molar ratios decrease. For (Pb_x_Ca_1−x_)_5_(PO_4_)_3_(OH) with X_Pb_ ≥ 0.89, Pb^2+^ and PO_4_^3−^ ions are partially re-absorbed from the solution onto the (Pb_x_Ca_1−x_)_5_(PO_4_)_3_(OH) surface when an initial part of (Pb_x_Ca_1−x_)_5_(PO_4_)_3_(OH) dissolves, the aqueous lead and phosphate concentrations decrease. Consequently, a new surface layer can form, which may have a different chemical composition from the bulk solid (Table 1). Due to the very low solubility of apatite, its dissolution is ever non-stoichiometric at the atomic level and includes a series of chemical reactions [7]. Finally, sorption and desorption of lead and phosphate reach a stable state. The aqueous lead and phosphate concentrations are almost invariable for the (Pb_x_Ca_1−x_)_5_(PO_4_)_3_(OH) dissolution in the acidic solution (initial pH of 2.00) at 25 °C from 5040 to 7200 h.

### Determination of solubility

The activities of the aqueous lead, calcium and phosphate species in the final equilibrated solutions (5040, 5760, 7200 h) are used to calculate the solubility products for the (Pb_x_Ca_1−x_)_5_(PO_4_)_3_OH solid solution.

The stoichiometric dissolution of the (Pb_x_Ca_1−x_)_5_(PO_4_)_3_OH solid solution and the release of Pb^2+^, Ca^2+^ and PO_4_^3−^ can be described according to Eq. (1). The solubility products (*K*_sp_) for (Pb_x_Ca_1−x_)_5_(PO_4_)_3_OH are equal to the ion activity products (IAP) at equilibrium:

7 $$K_{sp} = {\text{ IAP }} = \, \left\{ {{\text{Pb}}^{2 + } } \right\}^{{5{\text{x}}}} \left\{ {{\text{Ca}}^{2 + } } \right\}^{{5 - 5{\text{x}}}} \left\{ {{\text{PO}}_{4}^{3 - } } \right\}^{3} \left\{ {{\text{OH}}^{ - } } \right\}$$

where {} represents the thermodynamic activities of the aqueous Pb^2+^, Ca^2+^ and PO_4_^3−^.

The standard free energy of reaction (Δ*G*_*r*_^*o*^) can be calculated from *K*_sp_ at 298.15 K and 0.101 MPa (standard condition) by

8 $$\Delta G_{r}^{o} = \, - 5.708{ \log }K_{sp}$$

For Eq. (1),

9 $$\begin{aligned} \Delta G_{r}^{o} & = 5{\rm x} \Delta G_{f}^{o}[ {\rm Pb}^{2 + }] + ( 5 - 5{\rm x})\Delta G_{f}^{o} [{\rm Ca}^{2 + }] + 3\Delta G_{f}^{o} [{\rm PO}_{4}^{3 -}] \\ &\quad + \Delta G_{f}^{o}[{{\rm OH}}^{ - } ] - \Delta G_{f}^{o}[{{\rm OH}}^{-}] \\ & \quad -\Delta{G}_f^o[({{\rm Pb}}_{{\rm x}}{{\rm Ca}}_{1-{\rm x}})_5({\rm PO}_{4})_3{\rm OH}] \end{aligned}$$

Rearranging,

10 $$\begin{aligned}&\Delta G_{f}^{o} \left[ {\left( {{\text{Pb}}_{\text{x}} {\text{Ca}}_{{1 - {\text{x}}}} } \right)_{5} \left( {{\text{PO}}_{4} } \right)_{3} {\text{OH}}} \right] \, \\ & = 5{\text{x}}\Delta G_{f}^{o} \left[ {{\text{Pb}}^{2 + } } \right] + \left( {5 - 5{\text{x}}} \right)\Delta G_{f}^{o} \left[ {{\text{Ca}}^{2 + } } \right] \\ &\quad + 3\Delta G_{f}^{o} \left[ {{\text{PO}}_{4}^{3 - } } \right] + \Delta G_{f}^{o} [{\text{OH}}^{ - } ] \, - \Delta G_{r}^{o} \\ \end{aligned}$$

Table 2 lists the calculated solubility products (*K*_sp_) for (Pb_x_Ca_1−x_)_5_(PO_4_)_3_OH, as well as the pH, Pb, Ca and P analyses at 25 °C and an initial pH of 2.00. The solubility products (*K*_sp_) for the solid solution [(Pb_x_Ca_1−x_)_5_(PO_4_)_3_OH] decreased almost linearly with the increasing X_Pb_ from 10^−58.38±0.07^ to 10^−80.77±0.20^. Based on the following literature data obtained [39], Δ*G*_*f*_^*o*^[Pb^2+^] = −24.39 kJ/mol, Δ*G*_*f*_^*o*^[Ca^2+^] = −553.54 kJ/mol, Δ*G*_*f*_^*o*^[PO_4_^3−^] = −1018.8 kJ/mol, Δ*G*_*f*_^*o*^[OH^−^] = −157.3 kJ/mol, the free energies of formation, Δ*G*_*f*_^*o*^[(Pb_x_Ca_1−x_)_5_(PO_4_)_3_OH], were also calculated (Table 2). The solubility products (*K*_sp_) for (Pb_x_Ca_1−x_)_5_(PO_4_)_3_OH at 25 °C and an initial pH of 5.60 and 9.00 were also determined (Additional file 3: Appendix C).

**Tables 2:** Analytical data and solubility determination of the hydroxypyromorphite–hydroxyapatite solid solution [(Pb_x_Ca_1‒x_)_5_(PO_4_)_3_OH] (25 °C and an initial pH of 2.00)

Sample	Dissolution time (h)	pH	Concentration (mmol/L)			log*K*_sp_	Average log*K*_sp_	ΔG_*f*_^*o*^ (kJ/mol)	Average ΔG_*f*_^*o*^ (kJ/mol)
			Pb	Ca	P				
(Pb_0.00_Ca_1.00_)_5_(PO_4_)_3_OH	5040	4.75	0.00000	8.03	5.57	−58.46	−58.38	−6315.06	−6314.63
	5760	4.77	0.00000	8.03	5.59	−58.31		−6314.21	
	7200	4.76	0.00000	8.04	5.58	−58.38		−6314.61	
(Pb_0.10_Ca_0.90_)_5_(PO_4_)_3_OH	5040	4.55	0.00099	7.12	3.91	−62.39	−62.39	−6128.29	−6128.28
	5760	4.54	0.00111	7.14	3.95	−62.41		−6128.42	
	7200	4.55	0.00106	7.13	3.93	−62.36		−6128.12	
(Pb_0.20_Ca_0.80_)_5_(PO_4_)_3_OH	5040	4.46	0.00153	6.85	3.33	−64.99	−65.19	−5850.90	−5852.04
	5760	4.39	0.00172	6.85	3.36	−65.42		−5853.34	
	7200	4.44	0.00153	6.67	3.32	−65.16		−5851.87	
(Pb_0.30_Ca_0.70_)_5_(PO_4_)_3_OH	5040	4.36	0.00149	6.61	3.30	−67.59	−67.43	−5601.14	−5600.22
	5760	4.39	0.00171	6.62	3.32	−67.27		−5599.35	
	7200	4.37	0.00170	6.60	3.31	−67.42		−5600.17	
(Pb_0.41_Ca_0.59_)_5_(PO_4_)_3_OH	5040	4.45	0.00152	6.60	2.98	−69.07	−69.18	−5318.58	−5319.18
	5760	4.43	0.00151	6.60	3.02	−69.19		−5319.28	
	7200	4.42	0.00152	6.61	3.00	−69.27		−5319.69	
(Pb_0.51_Ca_0.49_)_5_(PO_4_)_3_OH	5040	4.43	0.00211	6.07	1.79	−71.35	−71.42	−5039.35	−5039.76
	5760	4.42	0.00215	6.07	1.79	−71.40		−5039.60	
	7200	4.40	0.00215	6.07	1.81	−71.52		−5040.33	
(Pb_0.61_Ca_0.39_)_5_(PO_4_)_3_OH	5040	4.32	0.00386	5.45	1.01	−73.84	−73.86	−4816.62	−4816.77
	5760	4.34	0.00401	5.39	1.01	−73.64		−4815.53	
	7200	4.28	0.00391	5.38	1.00	−74.10		−4818.15	
(Pb_0.69_Ca_0.31_)_5_(PO_4_)_3_OH	5040	4.30	0.00224	6.18	2.24	−75.01	−74.94	−4528.61	−4528.24
	5760	4.33	0.00230	6.17	2.28	−74.73		−4527.06	
	7200	4.28	0.00231	6.16	2.26	−75.08		−4529.04	
(Pb_0.80_Ca_0.20_)_5_(PO_4_)_3_OH	5040	4.12	0.00495	5.85	1.38	−77.40	−77.52	−4334.29	−4334.94
	5760	4.11	0.00468	5.89	1.40	−77.55		−4335.13	
	7200	4.09	0.00487	5.85	1.42	−77.59		−4335.39	
(Pb_0.89_Ca_0.11_)_5_(PO_4_)_3_OH	5040	3.41	0.11486	5.11	0.101	−81.11	−81.16	−4061.97	−4062.27
	5760	3.35	0.11197	5.00	0.107	−81.49		−4064.16	
	7200	3.36	0.13127	5.06	0.128	−80.88		−4060.67	
(Pb_1.00_Ca_0.00_)_5_(PO_4_)_3_OH	5040	2.63	3.75579	0.00	0.049	−80.96	−80.77	−3797.78	−3796.71
	5760	2.67	3.64382	0.00	0.046	−80.79		−3796.79	
	7200	2.68	3.70270	0.00	0.050	−80.57		−3795.55	

The average *K*_sp_ values were estimated for hydroxypyromorphite [Pb_5_(PO_4_)_3_OH] of 10^−80.77±0.20^ (10^−80.57^–10^−80.96^) at 25 °C, for Ca_5_(PO_4_)_3_OH of 10^−58.38^ (10^−58.31^–10^−58.46^) at 25 °C. The corresponding Gibbs free energies of formation (Δ*G*_*f*_^*o*^) were determined to be −3796.71 and −6314.63 kJ/mol.

The average *K*_sp_ for hydroxypyromorphite [Pb_5_(PO_4_)_3_OH] of 10^−80.77±0.20^ is comparable with the value reported for lead chloropyromorphite [Pb_5_(PO_4_)_3_Cl] of 10^−83.61^ [6]. Whereas, the Gibbs free energy of formation (Δ*G*_*f*_^*o*^) for hydroxypyromorphite [Pb_5_(PO_4_)_3_OH] of −3796.71 kJ/mol is lower than −3773.968 kJ/mol that was calculated from the *K*_sp_ of 10^−76.8^ for hydroxypyromorphite [Pb_5_(PO_4_)_3_OH] [38]. The average *K*_sp_ for calcium hydroxyapatite [Ca_5_(PO_4_)_3_OH] was calculated to be 10^−58.38±0.07^ (10^−58.31^–10^−58.46^) at 25 °C in in the present work. Various *K*_sp_ values for Ca_5_(PO_4_)_3_OH are reported in literatures, e.g., 10^−59^ [40], 10^−58.3^ [41], 10^−57^ [42], 10^−58±1^ [26], and 10^−57.72^ [43]. The large discrepancies in *K*_sp_ values may be caused by the differences in experimental conditions [7].

In comparison, the average *K*_sp_ 10^−80.77±0.20^ for Pb_5_(PO_4_)_3_OH is approximately 23.77–21.77 log units lower than 10^−57^–10^−59^ for Ca_5_(PO_4_)_3_OH, i.e., Pb_5_(PO_4_)_3_OH is extremely less soluble than Ca_5_(PO_4_)_3_OH, which shows that it is favorable for the transformation of Ca_5_(PO_4_)_3_OH to Pb_5_(PO_4_)_3_OH in presence of aqueous Pb^2+^ [44]. In the amendments of lead-contaminated soils with natural and synthetic phosphates, it is found that earlier dissolution of calcium hydroxyapatite [Ca_5_(PO_4_)_3_OH] can cause the following precipitation of the lead-bearing hydroxypyromorphite [Pb_5_(PO_4_)_3_OH] [19, 21–23]. Ca_5_(PO_4_)_3_OH dissolves continuously as the result of forming less soluble Pb_5_(PO_4_)_3_OH [41]. The transport-controlled Ca_5_(PO_4_)_3_OH dissolution can provide PO_4_^3−^ for the Pb_5_(PO_4_)_3_OH precipitation, which in turn consumes aqueous Pb^2+^ [44].

### Saturation index for calcium and lead hydroxyapatite

**Fig. 7:**
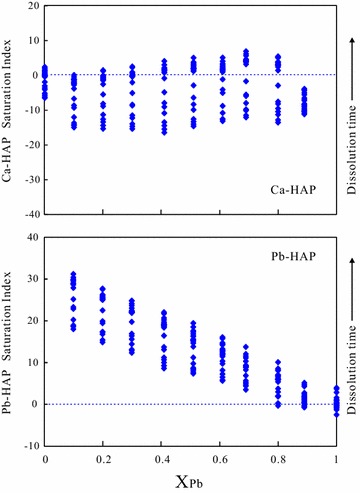
Calculated saturation indices for Pb-HAP and Ca-HAP

Thermodynamic analyses can be carried out first by supposing the potential pure-phase equilibrium relationships [45]. The saturation index (SI = log IAP/*K*_sp_) could be used to assess the pure-phase equilibrium, where IAP is the ion activity product ({Pb^2+^}^5^{PO_4_^3−^}^3^{OH^−^} or {Ca^2+^}^5^{PO_4_^3−^}^3^{OH^−^}) and *K*_sp_ is the solubility product of the pure-phase. If SI is close to zero, the solution is saturated with the solid; if SI is positive, the solution is supersaturated with the solid; and if SI is negative, the solution is undersaturated with the solid. The SI calculated for Ca-HAP [Ca_5_(PO_4_)_3_OH] has an obvious difference in the variational trend from that for Pb-HAP [Pb_5_(PO_4_)_3_OH] (Fig. 7). The maximum saturated index (SI) values for Ca_5_(PO_4_)_3_OH appeared at the Pb/(Pb + Ca) molar ratio (X_Pb_) of 0.69 [(Pb_0.69_Ca_0.31_)_5_(PO_4_)_3_OH]. The aqueous solutions are supersaturated with Ca_5_(PO_4_)_3_OH to the end of the dissolution experiment with (Pb_x_Ca_1−x_)_5_(PO_4_)_3_OH (X_Pb_ = 0.10–0.80). The aqueous solutions are considerably supersaturated with Pb_5_(PO_4_)_3_OH at the end of the experiment for all (Pb_x_Ca_1−x_)_5_(PO_4_)_3_OH solids. Generally, the SI values for Pb_5_(PO_4_)_3_OH decrease lineally with the increasing X_Pb_ of (Pb_x_Ca_1−x_)_5_(PO_4_)_3_OH. The dissolution–recrystallization can happen during the interaction between (Pb_x_Ca_1−x_)_5_(PO_4_)_3_OH and aqueous solution, and the less soluble component Pb-HAP [Pb_5_(PO_4_)_3_OH] tends to distribute preferentially towards the solid phase [25, 46, 47].

### Lippmann diagram

#### Construction of the Lippmann diagram

The solid solution–aqueous solution (SSAS) interaction plays an important role in the geochemical processes in water, rock and soil. However, the thermodynamic data about SSAS systems are still scarcely available, although the method to describe reaction paths and end points of equilibrium in SSAS systems has been discussed broadly [25, 45–52].

The sum of the partial activity products of the two endmembers can be defined as the “total activity product” ΣΠ_SS_ of the solid solution [30]. The Lippmann’s “*solidus*” relation expresses the total activity product at thermodynamic equilibrium (ΣΠ_eq_) as a function of the solid composition, and the Lippmann’s “*solutus*” relation is defined by expressing the total activity product at thermodynamic equilibrium (ΣΠ_eq_) as a function of the aqueous solution composition. The Lippmann diagram is a phase diagram that presents graphically the “*solidus*” and “*solutus*” relation.

When several sites for one formula unit of the substituting ions exist, the relationship between the component activities and the molar ratios of the substituting ions can simply be described by transforming it to a “one-substituting-ion” formula. For the solid solution (Pb_x_Ca_1‒x_)_5_(PO_4_)_3_OH, its formula unit can be redefined as (Pb_x_Ca_1‒x_)(PO_4_)_3/5_OH_1/5_, the formula units of the endmembers Pb_5_(PO_4_)_3_OH and Ca_5_(PO_4_)_3_OH can be redefined as Pb(PO_4_)_3/5_OH_1/5_ and Ca(PO_4_)_3/5_OH_1/5_, respectively.

In constructing the Lippmann diagram, the total solubility product (ΣΠ) for (Pb_x_Ca_1‒x_)(PO_4_)_3/5_OH_1/5_ can be expressed as [47]:

11 $$\begin{aligned} &\Sigma \Pi_{{ ( {\text{Pb}}_{\text{x}} {\text{Ca}}_{{ 1 { - }{\text{x}}}} ) ( {\text{PO}}_{ 4} )_{ 3 / 5} {\text{OH}}_{ 1 / 5} }} \\ &= \left( {\left\{ {{\text{Pb}}^{2 + } } \right\} + \{ {\text{Ca}}^{2 + } \} } \right)\left\{ {{\text{PO}}_{4}^{3 - } } \right\}^{3/5} \left\{ {{\text{OH}}^{ - } } \right\}^{1/5} \\ \; & = {\text{K}}_{{{\text{Pb(PO}}_{ 4} )_{ 3 / 5} {\text{OH}}_{ 1 / 5} }} {\text{X}}_{{{\text{Pb(PO}}_{ 4} )_{ 3 / 5} {\text{OH}}_{ 1 / 5} }} \gamma_{{{\text{Pb(PO}}_{ 4} )_{ 3 / 5} {\text{OH}}_{ 1 / 5} }} \\ &\quad + {\text{K}}_{{{\text{Ca(PO}}_{ 4} )_{ 3 / 5} {\text{OH}}_{ 1 / 5} }} {\text{X}}_{{{\text{Ca(PO}}_{ 4} )_{ 3 / 5} {\text{OH}}_{ 1 / 5} }} \gamma_{{{\text{Ca(PO}}_{ 4} )_{ 3 / 5} {\text{OH}}_{ 1 / 5} }} \\ \end{aligned}$$

where {} designates aqueous activity. ${\text{X}}_{{{\text{Pb(PO}}_{ 4} )_{ 3 / 5} {\text{OH}}_{ 1 / 5} }}$ and ${\text{X}}_{{{\text{Ca(PO}}_{ 4} )_{ 3 / 5} {\text{OH}}_{ 1 / 5} }}$, $\gamma_{{{\text{Pb(PO}}_{ 4} )_{ 3 / 5} {\text{OH}}_{ 1 / 5} }}$ and $\gamma_{{{\text{Ca(PO}}_{ 4} )_{ 3 / 5} {\text{OH}}_{ 1 / 5} }}$, ${\text{K}}_{{{\text{Pb(PO}}_{ 4} )_{ 3 / 5} {\text{OH}}_{ 1 / 5} }}$ and ${\text{K}}_{{{\text{Ca(PO}}_{ 4} )_{ 3 / 5} {\text{OH}}_{ 1 / 5} }}$ are the mole fractions (x, 1−x), the activity coefficients and the thermodynamic solubility products of Pb(PO_4_)_3/5_OH_1/5_ and Ca(PO_4_)_3/5_OH_1/5_ in the solid solution (Pb_x_Ca_1−x_)(PO_4_)_3/5_OH_1/5_. This *solidus* equation expresses all possible thermodynamic saturation states for (Pb_x_Ca_1−x_)(PO_4_)_3/5_OH_1/5_ based on the solid component [53].

The *solutus* relation can be expressed as [47]:

12 $$\begin{aligned} &{{\Sigma }}\Pi_{{ ( {\text{Pb}}_{\text{x}} {\text{Ca}}_{{ 1- {\text{x}}}} ) ( {\text{PO}}_{ 4} )_{ 3 / 5} {\text{OH}}_{ 1 / 5} }} \\ & = \frac{1}{{\frac{{{\text{X}}_{{{\text{Pb}}^{ 2+ } , {\text{aq}}}} }}{{{\text{K}}_{{{\text{Pb(PO}}_{ 4} )_{ 3 / 5} {\text{OH}}_{ 1 / 5} }} \gamma_{{{\text{Pb(PO}}_{ 4} )_{ 3 / 5} {\text{OH}}_{ 1 / 5} }} }} + \frac{{{\text{X}}_{{{\text{Ca}}^{ 2+ } , {\text{aq}}}} }}{{{\text{K}}_{{{\text{Ca(PO}}_{ 4} )_{ 3 / 5} {\text{OH}}_{ 1 / 5} }} \gamma_{{{\text{Ca(PO}}_{ 4} )_{ 3 / 5} {\text{OH}}_{ 1 / 5} }} }}}} \end{aligned}$$

where ${\text{X}}_{{{\text{Pb}}_{{}}^{2 + } ,{\text{aq}}}}$ and ${\text{X}}_{{{\text{Ca}}_{{}}^{2 + } ,{\text{aq}}}}$ are the activity fractions for {Pb^2+^} and {Ca^2+^} in the aqueous phase, respectively. This equation expresses all possible thermodynamic saturation states for (Pb_x_Ca_1−x_)(PO_4_)_3/5_OH_1/5_ based on the aqueous composition [53].

The total solubility product ($\Sigma \Pi_{{ ( {\text{Pb}}_{\text{x}} {\text{Ca}}_{{ 1- {\text{x}}}} ) ( {\text{PO}}_{ 4} )_{ 3 / 5} {\text{OH}}_{ 1 / 5} }}$) for (Pb_x_Ca_1−x_)(PO_4_)_3/5_OH_1/5_ at stoichiometric saturation can be expressed as:

13 $$\begin{aligned} &{{\Sigma }}\Pi_{{ ( {\text{Pb}}_{\text{x}} {\text{Ca}}_{{ 1 -{\text{ x}}}} ) ( {\text{PO}}_{ 4} )_{ 3 / 5} {\text{OH}}_{ 1 / 5} }} \\ & = \frac{{{\text{K}}_{{ ( {\text{Pb}}_{\text{x}} {\text{Ca}}_{{ 1 - {\text{ x}}}} ) ( {\text{PO}}_{ 4} )_{ 3 / 5} {\text{OH}}_{ 1 / 5} }} }}{{({\text{X}}_{{{\text{Pb}}^{ 2+ } , {\text{aq}}}} )^{{{\text{X}}_{{{\text{Pb(PO}}_{ 4} )_{ 3 / 5} {\text{OH}}_{ 1 / 5} }} }} ({\text{X}}_{{{\text{Ca}}^{ 2+ } , {\text{aq}}}} )^{{{\text{X}}_{{{\text{Ca(PO}}_{ 4} )_{ 3 / 5} {\text{OH}}_{ 1 / 5} }} }} }}\end{aligned}$$

where ${\text{K}}_{{ ( {\text{Pb}}_{\text{x}} {\text{Ca}}_{{ 1- {\text{x}}}} ) ( {\text{PO}}_{ 4} )_{ 3 / 5} {\text{OH}}_{ 1 / 5} }}^{{}}$, {Pb^2+^}^x^{Ca^2+^}^(1−x)^{PO_4_^3−^}^3/5^{OH^−^}^1/5^, is the stoichiometric saturation constant for (Pb_x_Ca_1−x_)(PO_4_)_3/5_OH_1/5_.

The total solubility products for the stoichiometric saturation with Pb(PO_4_)_3/5_OH_1/5_ and Ca(PO_4_)_3/5_OH_1/5_, ${{\Sigma }}\Pi_{{{\text{Pb(PO}}_{ 4} )_{ 3 / 5} {\text{OH}}_{ 1 / 5} }}$ and ${{\Sigma }}\Pi_{{{\text{Ca(PO}}_{ 4} )_{ 3 / 5} {\text{OH}}_{ 1 / 5} }}$, can be expressed by their solubility products ${\text{K}}_{{{\text{Pb(PO}}_{ 4} )_{ 3 / 5} {\text{OH}}_{ 1 / 5} }}$ and ${\text{K}}_{{{\text{Ca(PO}}_{ 4} )_{ 3 / 5} {\text{OH}}_{ 1 / 5} }}$, respectively:

14 $${{\Sigma }}\Pi_{{{\text{Pb(PO}}_{ 4} )_{ 3 / 5} {\text{OH}}_{ 1 / 5} }} = \frac{{{\text{K}}_{{{\text{Pb(PO}}_{ 4} )_{ 3 / 5} {\text{OH}}_{ 1 / 5} }} }}{{({\text{X}}_{{{\text{Pb}}^{ 2+ } , {\text{aq}}}} )^{{{\text{X}}_{{{\text{Pb(PO}}_{ 4} )_{ 3 / 5} {\text{OH}}_{ 1 / 5} }} }} }}$$

15 $${{\Sigma }}\Pi_{{{\text{Ca(PO}}_{ 4} )_{ 3 / 5} {\text{OH}}_{ 1 / 5} }} = \frac{{{\text{K}}_{{{\text{Ca(PO}}_{ 4} )_{ 3 / 5} {\text{OH}}_{ 1 / 5} }} }}{{({\text{X}}_{{{\text{Ca}}^{ 2+ } , {\text{aq}}}} )^{{{\text{X}}_{{{\text{Ca(PO}}_{ 4} )_{ 3 / 5} {\text{OH}}_{ 1 / 5} }} }} }}$$

The “total solubility product ${{\Sigma }}\Pi_{{ ( {\text{Pb}}_{\text{x}} {\text{Ca}}_{{ 1- {\text{x}}}} )_{ 5} ( {\text{PO}}_{ 4} )_{ 3} {\text{OH}}}}$” for the Pb–Ca-HAP solid solution with the formula unit of (Pb_x_Ca_1−x_)_5_(PO_4_)_3_OH can be calculated from the “total solubility product ${\Sigma}\Pi_{{ ( {\text{Pb}}_{\text{x}} {\text{Ca}}_{{ 1- {\text{x}}}} ) ( {\text{PO}}_{ 4} )_{ 3 / 5} {\text{OH}}_{ 1 / 5} }}$” by

16 $$\begin{aligned} &{\Sigma \Pi }_{{ ( {\text{Pb}}_{\text{x}} {\text{Ca}}_{{ 1- {\text{x}}}} )_{ 5} ( {\text{PO}}_{ 4} )_{ 3} {\text{OH}}}} \\ & = \left( {\left\{ {{\text{Pb}}^{2 + } } \right\} + \{ {\text{Ca}}^{2 + } \} } \right)^{5} \left\{ {{\text{PO}}_{4}^{3 - } } \right\}^{3} \left\{ {{\text{OH}}^{ - } } \right\} \\ & = \, \left[ {\left( {\left\{ {{\text{Pb}}^{2 + } } \right\} + \{ {\text{Ca}}^{2 + } \} } \right)\left\{ {{\text{PO}}_{4}^{3 - } } \right\}^{3/5} \left\{ {{\text{OH}}^{ - } } \right\}^{1/5} } \right]^{5} \\ & = \, \left[ {{\Sigma}\Pi_{{ ( {\text{Pb}}_{\text{x}} {\text{Ca}}_{{ 1- {\text{x}}}} ) ( {\text{PO}}_{ 4} )_{ 3 / 5} {\text{OH}}_{ 1 / 5} }} } \right]^{5}\end{aligned}$$

**Fig. 8:**
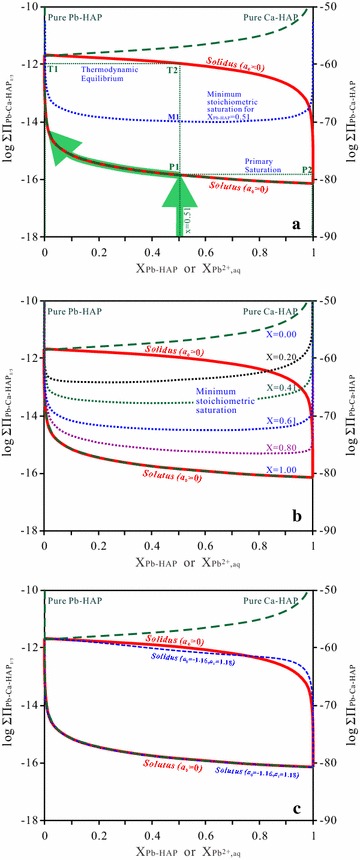
Lippmann diagrams for dissolution of the hydroxypyromorphite–hydroxyapatite solid solution [(Pb_x_Ca_1−x_)_5_(PO_4_)_3_OH] at 25 °C and an initial pH of 2.00. **a** Assuming an ideal solid-solution. Hypothetical partial-equilibrium reaction path for the dissolution of the solid phase (Pb_x_Ca_1−x_)_5_(PO_4_)_3_OH (x = 0.51) is drawn in the *arrowed solid lines*. *Solid arrows* show primary saturation states; **b** *Long-dotted* or *dashed curves* depict the series of possible stoichiometric saturation states for the (Pb_x_Ca_1−x_)_5_(PO_4_)_3_OH solid solution (x = 0.00, 0.20, 0.41, 0.61, 0.80 and 1.00); **c** Assuming a non-ideal solid-solution based on the estimated Guggenheim parameters *a*_*0*_ = −1.16 and *a*_*1*_ = 1.18

Finally, the Lippmann diagram for the Pb–Ca-HAP solid solution as (Pb_x_Ca_1−x_)_5_(PO_4_)_3_OH can be constructed by plotting the *solidus* and *solutus* as log ${{\Sigma }}\Pi_{{ ( {\text{Pb}}_{\text{x}} {\text{Ca}}_{{ 1 -\text{ }{\text{x}}}} )_{ 5} ( {\text{PO}}_{ 4} )_{ 3} {\text{OH}}}}$ (or log[${{\Sigma }}\Pi_{{ ( {\text{Pb}}_{\text{x}} {\text{Ca}}_{{ 1 -{\text{ x}}}} ) ( {\text{PO}}_{ 4} )_{ 3 / 5} {\text{OH}}_{ 1 / 5} }}$]^5^) on the ordinate vs two superimposed aqueous and solid phase mole fraction scales on the abscissa (Fig. 8a). The curves are calculated from the solubility products for Pb_5_(PO_4_)_3_OH of 10^−80.77^ and Ca_5_(PO_4_)_3_OH of 10^−58.38^ of the present work.

In Fig. 8a, the *solutus* curve of the Lippmann diagram is near the curve for the pure endmember Pb-HAP [Pb_5_(PO_4_)_3_OH, x = 1.00]. For comparison with the Lippmann *solutus* curve, some hypothetical stoichiometric saturation curves for (Pb_x_Ca_1−x_)_5_(PO_4_)_3_OH (x = 0.00, 0.20, 0.41, 0.61, 0.80 and 1.00) are also calculated and plotted in Fig. 8b. The Lippmann *solutus* curve and the stoichiometric saturation curves are similar in shape, and the stoichiometric saturation curves are close to the *solutus* curve as the solid-components are near the less soluble endmember Pb-HAP [Pb_5_(PO_4_)_3_OH] [46]. Because of the large difference between the solubility products of Pb_5_(PO_4_)_3_OH and Ca_5_(PO_4_)_3_OH, the stoichiometric saturation for the sparingly soluble Pb-HAP [Pb_5_(PO_4_)_3_OH] is very close to the Lippmann *solutus* curve [46].

**Fig. 9:**
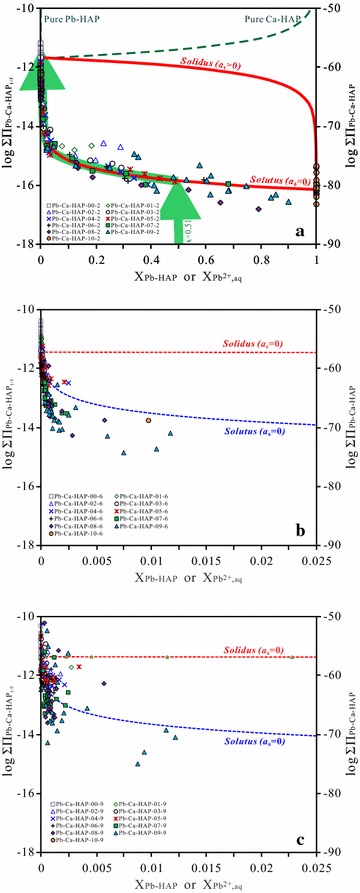
Plotting of the experimental data on the Lippmann diagrams for dissolution of the hydroxypyromorphite–hydroxyapatite solid solution [(Pb_x_Ca_1−x_)_5_(PO_4_)_3_OH]. **a** 25 °C and an initial pH of 2.00, the *arrows* indicated the evolution directions of the aqueous solution during the solid solution–aqueous solution interaction; **b** 25 °C and an initial pH of 5.60; **c** 25 °C and an initial pH of 9.00

The hypothetical reaction path for (Pb_0.51_Ca_0.49_)_5_(PO_4_)_3_OH is also calculated and plotted in comparison with the Lippmann *solutus* and *solidus* curves for the Pb–Ca-HAP solid solution [(Pb_x_Ca_1−x_)_5_(PO_4_)_3_OH] (Figs. 8a, 9a). In the beginning, the (Pb_0.51_Ca_0.49_)_5_(PO_4_)_3_OH solid dissolves stoichiometrically in aqueous solution and its reaction path moves up vertically to the Lippmann *solutus* curve, which shows that the mole fraction for the aqueous solution is the same as the initial solid solution component [53]. And then, the (Pb_0.51_Ca_0.49_)_5_(PO_4_)_3_OH solid dissolves non-stoichiometrically and the reaction path moves along the *solutus* curve towards the more soluble endmember Ca-HAP. This is in accordance with the result of the dissolution experiment for (Ba,Sr)SO_4_ [46]. In the Lippmann diagram for the (Ba,Sr)SO_4_–H_2_O system, the reaction pathways show initial congruent dissolution up to the *solutus* curve, followed by incongruent dissolution along the *solutus* curve towards the more soluble endmember SrSO_4_ [46]. There are two possible limiting reaction paths [45], i.e., the stoichiometric dissolution of (Pb_x_Ca_1−x_)_5_(PO_4_)_3_OH up to the first point of saturation (primary saturation) with a secondary solid phase, either a solid-solution phase or a pure solid phase, and the following non-stoichiometric dissolution with an increasing substitution reaction [45, 46]. For the solid solution (Pb_x_Ca_1‒x_)_5_(PO_4_)_3_OH, this exchange reaction could be

17 $$\left( {{\text{Pb}}_{\text{x}} {\text{Ca}}_{{1 - {\text{x}}}} } \right)_{5} \left( {{\text{PO}}_{4} } \right)_{3} {\text{OH }} + \, 5\Delta i{\text{Pb}}^{2 + } = \, \left( {{\text{Pb}}_{{{\text{x}} + \Delta i}} {\text{Ca}}_{{1 - {\text{x}} - \Delta i}} } \right)_{5} \left( {{\text{PO}}_{4} } \right)_{3} {\text{OH }} + \, 5\Delta i{\text{Ca}}^{2 + }$$

This reaction path follows the Lippmann *solutus* curve that can present some primary saturation states. Consequently, the sparingly soluble endmember Pb-HAP [Pb_5_(PO_4_)_3_OH] will be gradually enriched in the solid phases, whereas the aqueous solution will become progressively rich in Ca^2+^ when an equilibrium or a stable state is attained [46]. In the stoichiometric dissolution, the solid component does not change, but the activity ratios {Pb^2+^}/({Pb^2+^}+{Ca^2+^}) in the aqueous phase may vary as the reaction progresses.

The activity coefficients of Pb(PO_4_)_3/5_OH_1/5_ and Ca(PO_4_)_3/5_OH_1/5_ in the solid solution (Pb_x_Ca_1−x_)(PO_4_)_3/5_OH_1/5_ can be approximated using the Redlich and Kister equation. The Guggenheim coefficients *a*_*0*_ and *a*_*1*_ were estimated by fitting the solubility products (${\text{K}}_{{ ( {\text{Pb}}_{\text{x}} {\text{Ca}}_{{ 1- {\text{x}}}} ) ( {\text{PO}}_{ 4} )_{ 3 / 5} {\text{OH}}_{ 1 / 5} }}$) as a function of the solid components to Eq. (18).

18 $$\begin{aligned} &{ \ln }\;{\text{K}}_{{ ( {\text{Pb}}_{\text{x}} {\text{Ca}}_{{ 1- {\text{x}}}} ) ( {\text{PO}}_{ 4} )_{ 3 / 5} {\text{OH}}_{ 1 / 5} }} \\ &= {\text{x}}\left( {1 - {\text{x}}} \right)a_{0} + {\text{ x}}\left( {1 - {\text{x}}} \right)\left( {{\text{x}} - \left( {1 - {\text{x}}} \right)} \right)a_{1} \\ & \quad + \, \left( {1 - {\text{x}}} \right){ \ln }\left[{\text{K}}_{{{\text{Ca(PO}}_{ 4} )_{ 3 / 5} {\text{OH}}_{ 1 / 5} }} \left( {1 - x} \right)\right] \\ & \quad + {\text{ xln}} \left[{\text{K}}_{{{\text{Pb(PO}}_{ 4} )_{ 3 / 5} {\text{OH}}_{ 1 / 5} }} {\text{x}})\right] \end{aligned}$$

where ${\text{K}}_{{{\text{Pb(PO}}_{ 4} )_{ 3 / 5} {\text{OH}}_{ 1 / 5} }}$ and ${\text{K}}_{{{\text{Ca(PO}}_{ 4} )_{ 3 / 5} {\text{OH}}_{ 1 / 5} }}$ are the solubility products of Pb(PO_4_)_3/5_OH_1/5_ and Ca(PO_4_)_3/5_OH_1/5_, respectively.

The Lippmann diagram for the non-ideal solid solution (Pb_x_Ca_1−x_)_5_(PO_4_)_3_OH was calculated and constructed with the estimated Guggenheim parameters *a*_*0*_ = −1.16 and *a*_*1*_ = 1.18 (Fig. 8c). The diagram in the Fig. 8c is a typical Lippmann diagram for the solid solution with a negative enthalpy of mixing. The stoichiometric saturation curve for pure Pb-HAP [Pb_5_(PO_4_)_3_OH] is similar to the Lippmann *solutus* curve and close to the *solutus* curve as the solid components are near the sparingly soluble Pb-HAP [46]. Due to the large difference between the solubility products of the two pure endmembers Pb-HAP (10^−80.77^) and Ca-HAP (10^−58.38^), the Lippmann *solutus* curve for the non-ideal solid solution (Pb_x_Ca_1−x_)_5_(PO_4_)_3_OH is very close to the curve for the sparingly soluble endmember and the Lippmann *solutus* curve for the ideal solid solution (Pb_x_Ca_1−x_)_5_(PO_4_)_3_OH.

The solid solution (Pb_x_Ca_1−x_)_5_(PO_4_)_3_OH can be treated as an ideal one in constructing the Lippmann diagram because the Lippmann *solutus* position is insensitive to the excess Gibbs free energy of mixing, although the position of the Lippmann *solidus* can be obviously affected [46] (Fig. 8c). This phenomenon is observed in all SSAS systems with a large difference between the solubility products of two endmembers, for which the excess Gibbs free energy of mixing has a small effect on the Lippmann *solutus* position [46]. The Lippmann diagram constructed for (Pb_x_Ca_1−x_)_5_(PO_4_)_3_OH as a non-ideal solid solution is very similar to the diagram for (Pb_x_Ca_1−x_)_5_(PO_4_)_3_OH as the ideal solid solution only with the difference of a slight upward convexity of the *solidus* curve at high X_Pb_ or a slight downward concavity of the *solidus* curve at low X_Pb_ [25], which indicates that the SSAS interaction for the solid solution (Pb_x_Ca_1−x_)_5_(PO_4_)_3_OH is not greatly affected by its non-ideality.

#### Solid-solution aqueous-solution reaction paths

The experimental data are plotted as ({Pb^2+^}+{Ca^2+^})^5^{PO_4_^3−^}^3^{OH^−^} vs. X_Pb,aq_ in the Lippmann diagram for the ideal solid-solution (Pb_x_Ca_1‒x_)_5_(PO_4_)_3_OH (Fig. 9a, b, c). The saturation curves for Pb_5_(PO_4_)_3_(OH) (x = 1.00) and Ca_5_(PO_4_)_3_(OH) (x = 0.00) are also plotted in the diagram. In general, the positions of the data points on the Lippmann diagram are related to the rates of dissolution and precipitation, the aqueous speciation, and the degree of the formation of secondary phases. When (Pb_x_Ca_1−x_)_5_(PO_4_)_3_OH dissolves in solution, the aqueous Pb^2+^ is converted into PbOH^+^, Pb(OH)_2_^0^, Pb(OH)_3_^−^, Pb(OH)_4_^2−^, Pb_2_OH^3+^, Pb_3_(OH)_4_^2+^, Pb_4_(OH)_4_^4+^, PbHPO_4_^0^, PbH_2_PO_4_^+^ and PbP_2_O_7_^2−^, and aqueous Ca^2+^ is converted into CaOH^+^, CaHPO_4_, CaPO_4_^−^ and CaH_2_PO_4_^+^, aqueous PO_4_^3−^ is converted primarily into HPO_4_^2−^, H_2_PO_4_^−^, H_3_PO_4_^0^, CaHPO_4_, CaPO_4_^−^ and CaH_2_PO_4_^+^. The speciations can result in a smaller activity ratio of the aqueous Pb^2+^ to Ca^2+^. The speciations of the aqueous Pb^2+^ and Ca^2+^ are considered in plotting the experimental data on the Lippmann diagram by calculating the activities of Pb^2+^ and Ca^2+^ with PHREEQC.

For the (Pb_x_Ca_1−x_)_5_(PO_4_)_3_OH dissolution at 25 °C and an initial pH of 2.00, the plotting of the experimental data on the Lippmann diagram shows that the (Pb_0.51_Ca_0.49_)_5_(PO_4_)_3_(OH) solid dissolved in the aqueous solution stoichiometrically at the early stage and approached to the Lippmann *solutus* and the saturation curves for pure Pb-HAP [Pb_5_(PO_4_)_3_OH]. After 1 h dissolution, the aqueous solution was supersaturated with respect to (Pb_0.51_Ca_0.49_)_5_(PO_4_)_3_(OH) and Pb-HAP. After that, the X_Pb,aq_ decreased with the decreasing logΣΠ_SS_ value, and the data points moved along the Lippmann *solutus* curve from right to left (Fig. 9a), indicating that the reaction path for the solid dissolution includes an early stoichiometric dissolution up to the Lippmann *solutus* curve which is then followed by some possible substitution reactions [45, 46]. For the (Pb_x_Ca_1−x_)_5_(PO_4_)_3_OH dissolution at an initial pH of 5.60 or 9.00, the plotting of the experimental data on the Lippmann diagram illustrates that the X_Pb,aq_ values are significantly lower that X_Pb_ of the solids, which means that all solids dissolved in the aqueous solution non-stoichiometrically and approached to the Lippmann *solutus* and the saturation curves for pure Pb-HAP [Pb_5_(PO_4_)_3_OH] (Fig. 9b, c).

The results show a continuous increase of the Ca^2+^ ions in the aqueous phase and a continuous increase of the Pb-HAP [Pb_5_(PO_4_)_3_OH] component in the solid phase (Table 1; Fig. 9). A solid phase with a component near the pure Pb-HAP [Pb_5_(PO_4_)_3_OH] can form because of the very low solubility of Pb-HAP [Pb_5_(PO_4_)_3_OH] and the great supersaturation of the aqueous solution with Pb-HAP, and the relatively high solubility of Ca-HAP [Ca_5_(PO_4_)_3_OH] and the undersaturation of the aqueous solution with Ca-HAP.

The large difference between the solubility products of Pb_5_(PO_4_)_3_OH and Ca_5_(PO_4_)_3_OH can cause an preferential enrichment of the sparingly soluble Pb_5_(PO_4_)_3_OH in the solid phase [25, 51], i.e., a Pb-HAP-rich solid phase is to be in equilibrium with a Pb-poor aqueous phase or a Ca-HAP-poor solid phase in equilibrium with a Ca-rich aqueous phase. Therefore, it is practical to solidify/stabilize Pb-contaminated soils and Pb-containing hazardous wastes by using phosphates (apatites). Since lead hydroxyapatite [hydroxypyromorphite, Pb_5_(PO_4_)_3_(OH)] is stable and significantly less soluble than calcium hydroxyapatite [Ca_5_(PO_4_)_3_OH], it can be considered for safe disposal of industrial and mineral processing Pb-containing wastes and lead ions can be effectively removed from Pb-contaminated wastewaters by using hydroxyapatite.

## Conclusions

The characterization with XRD, FT-IR, SEM and TEM showed that the hydroxypyromorphite–hydroxyapatite solid solution [(Pb_x_Ca_1−x_)_5_(PO_4_)_3_(OH)] with apatite structure was not found to change obviously after dissolution except in some cases of the dissolution at the initial pH 2.00. In general, the final solution pHs decreased with the increasing Pb/(Pb + Ca) molar ratios (X_Pb_) of (Pb_x_Ca_1−x_)_5_(PO_4_)_3_(OH). The aqueous element concentrations were greatly affected by X_Pb_ during the dissolution. For the solids with high X_Pb_ [(Pb_0.89_Ca_0.11_)_5_(PO_4_)_3_OH], the aqueous Ca^2+^ concentrations increased gradually with the dissolution time and reached a stable state after 4320 h dissolution; the aqueous Pb^2+^ concentrations increased rapidly with time and reached a peak value after 240–720 h dissolution, and then decreased gradually and attained a stable state after 5040 h dissolution; the aqueous phosphate concentrations increased rapidly with time and achieved a peak value after 1–12 h dissolution, and then decreased gradually and attained a stable state after 2160 h dissolution.

For the solids with low X_Pb_ (0.00–0.80), the aqueous Ca^2+^ concentrations increased slowly with time and reached a peak value after 1200–1800 h dissolution, and then decreased slightly and were relatively stable after 4320 h dissolution; the aqueous Pb^2+^ concentrations increased quickly with time and reached a peak value after 1–12 h dissolution, and then decreased gradually and attained a stable state after 720–2160 h dissolution; the aqueous phosphate concentrations showed the same evolution trend as the aqueous Ca^2+^ concentrations. The dissolution process of (Pb_x_Ca_1−x_)_5_(PO_4_)_3_(OH) with high X_Pb_ (0.89–1.00) was different from that of (Pb_x_Ca_1−x_)_5_(PO_4_)_3_(OH) with low X_Pb_ (0.00–0.80), which was considered to be related to a small preference of larger Pb^2+^ to occupy the M(II) sites and smaller Ca^2+^ to occupy the M(I) sites in the apatite structure. For the dissolution of (Pb_x_Ca_1−x_)_5_(PO_4_)_3_(OH) with high X_Pb_ in the acidic solution, Pb^2+^, which occupied nearly all the M(2) sites, could be preferentially released because of the interaction of the solution H^+^ with the OH surrounding the M(2) atom. For the dissolution of (Pb_x_Ca_1−x_)_5_(PO_4_)_3_(OH) with low X_Pb_ in the acidic solution, Ca^2+^ in the M(2) sites was preferentially released with respect to Pb^2+^ in the M(2) sites.

The average *K*_sp_ values were estimated for hydroxypyromorphite [Pb_5_(PO_4_)_3_OH] of 10^−80.77^ (10^−80.57^–10^−80.96^) at 25 °C, for hydroxyapatite [Ca_5_(PO_4_)_3_OH] of 10^−58.38^ (10^−58.31^–10^−58.46^) at 25 °C, the Gibbs free energies of formation (Δ*G*_*f*_^*o*^) were determined to be −3796.71 and −6314.63 kJ/mol, respectively. The solubility of the solid solution (Pb_x_Ca_1−x_)_5_(PO_4_)_3_(OH) decreased with the increasing Pb/(Pb + Ca) molar ratios (X_Pb_) of (Pb_x_Ca_1−x_)_5_(PO_4_)_3_(OH). For the dissolution at 25 °C and an initial pH of 2.00, the experimental data plotted on the Lippmann diagram showed that the solid solution (Pb_x_Ca_1−x_)_5_(PO_4_)_3_(OH) dissolved congruently during the early stage of dissolution and moved gradually up to the Lippmann *solutus* curve, and then followed by incongruent dissolution and the data points moved along the Lippmann *solutus* curve from right to left, i.e., towards the more soluble endmember [Ca_5_(PO_4_)_3_OH]. The Pb-rich or Ca-poor (Pb_x_Ca_1−x_)_5_(PO_4_)_3_(OH) was in equilibrium with the Ca-rich aqueous solution.

## Additional files

10.1186/s12932-016-0034-8 Supplementary data—X-ray diffractograms (XRD) of the hydroxypyromorphite–hydroxyapatite solid solution [(Pb_x_Ca_1−x_)_5_(PO_4_)_3_(OH)] after dissolution at 25 ˚C and an initial pH of 5.60 and 9.00 for 300d.
10.1186/s12932-016-0034-8 Supplementary data—Change of the solution Pb/(Pb+Ca) molar ratios with time for the hydroxypyromorphite–hydroxyapatite solid solution [(Pb_x_Ca_1−x_)_5_(PO_4_)_3_(OH)] at 25 ˚C and an initial pH of 2.00, 5.60 and 9.00.
10.1186/s12932-016-0034-8 Supplementary data—Analytical data and solubility determination of the hydroxypyromorphite–hydroxyapatite solid solution [(Pb_x_Ca_1−x_)_5_(PO_4_)_3_OH] (25 ˚C, an initial pH of 5.60 and 9.00).
