# Supplementary material for: Characterization, dissolution and solubility of the hydroxypyromorphite–hydroxyapatite solid solution [(PbxCa1−x)5(PO4)3OH] at 25 °C and pH 2–9
Source: Geochem Trans. 2016 May 6;17:2. doi: 10.1186/s12932-016-0034-8 (PMC4858909; doi:10.1186/s12932-016-0034-8)
Supplement: Supplementary file 1 — 10.1186/s12932-016-0034-8 Supplementary data—X-ray diffractograms (XRD) of the hydroxypyromorphite–hydroxyapatite solid solution [(PbxCa1−x)5(PO4)3(OH)] after dissolution at 25 ˚C and an initial pH of 5.60 and 9.00 for 300d. [file 12932_2016_34_MOESM1_ESM.docx]

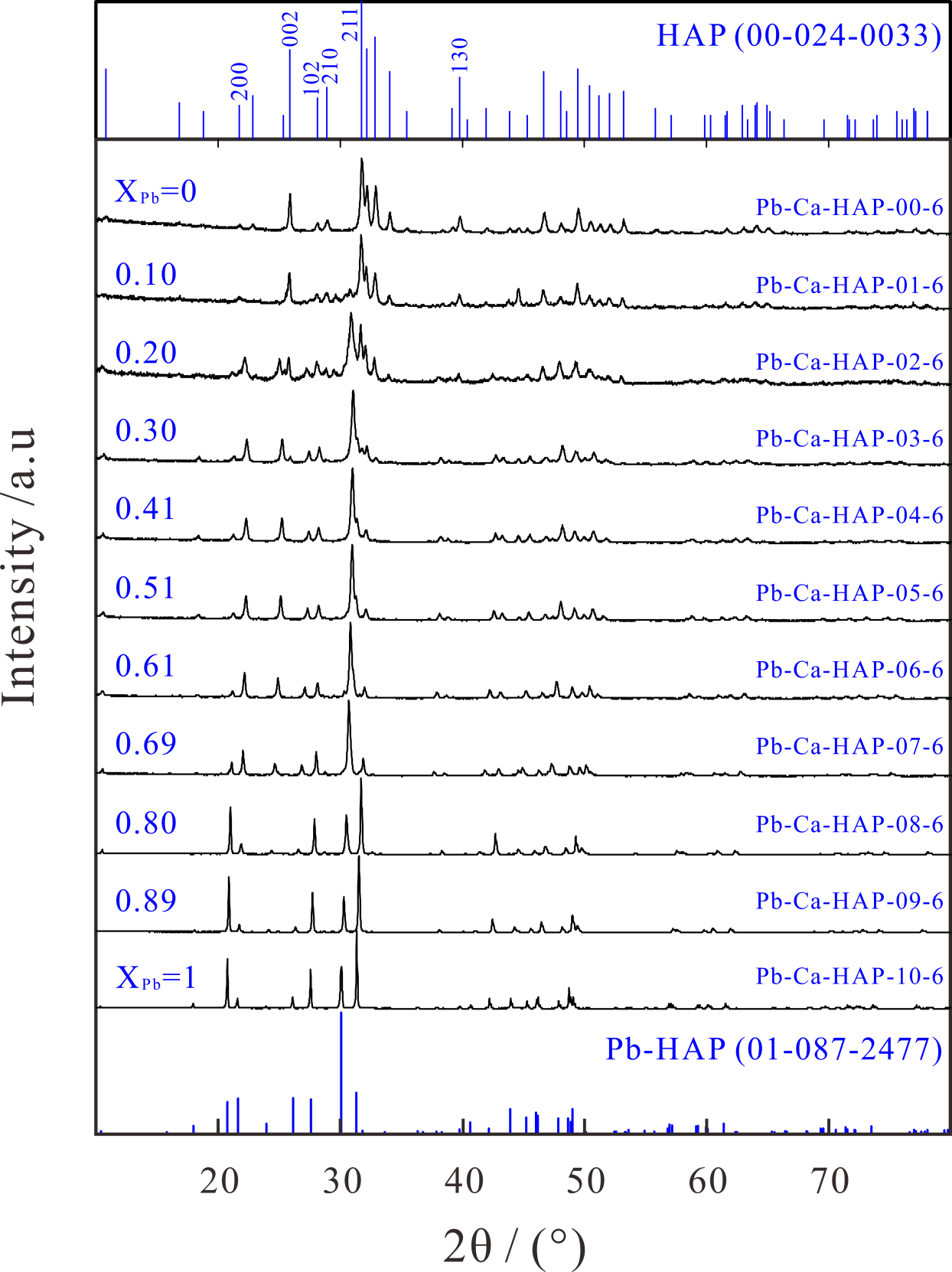


**Appendix A. Supplementary data --** X-ray diffractograms (XRD) of the hydroxypyromorphite–hydroxyapatite solid solution [(Pb_x_Ca_1‒x_)_5_(PO_4_)_3_(OH)] after dissolution at 25˚C and an initial pH of 5.6 for 300d.


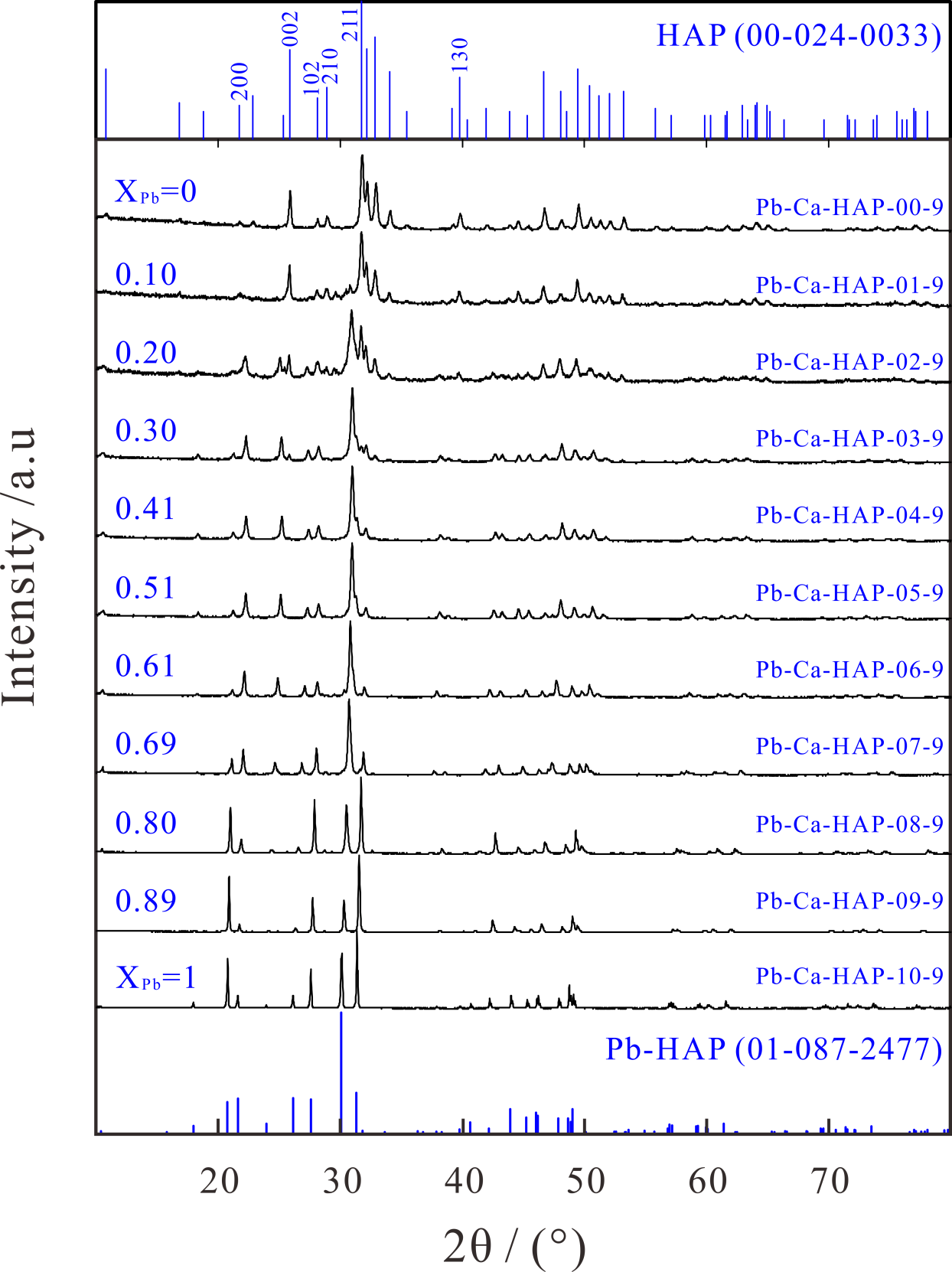


**Appendix A. Supplementary data --** X-ray diffractograms (XRD) of the hydroxypyromorphite–hydroxyapatite solid solution [(Pb_x_Ca_1‒x_)_5_(PO_4_)_3_(OH)] after dissolution at 25˚C and an initial pH of 9.0 for 300d.
