# Supplementary material for: Characterization, dissolution and solubility of the hydroxypyromorphite–hydroxyapatite solid solution [(PbxCa1−x)5(PO4)3OH] at 25 °C and pH 2–9
Source: Geochem Trans. 2016 May 6;17:2. doi: 10.1186/s12932-016-0034-8 (PMC4858909; doi:10.1186/s12932-016-0034-8)
Supplement: Supplementary file 3 — 10.1186/s12932-016-0034-8 Supplementary data—Analytical data and solubility determination of the hydroxypyromorphite–hydroxyapatite solid solution [(PbxCa1−x)5(PO4)3OH] (25 ˚C, an initial pH of 5.60 and 9.00). [file 12932_2016_34_MOESM3_ESM.doc]

**Appendix C-1. Supplementary data**

Analytical data and solubility determination of the hydroxypyromorphite–hydroxyapatite solid solution [(Pb_x_Ca_1‒x_)_5_(PO_4_)_3_OH]

(25˚C and an initial pH of 5.6)

| Sample | Dissolution time (h) | pH | Concentration (mmol/L) | | | log*K*_sp_ | Average log*K*_sp_ | ΔG*_f_^o^* (kJ/mol) | Average ΔG*_f_^o^* (kJ/mol) |
| --- | --- | --- | --- | --- | --- | --- | --- | --- | --- |
|  |  |  | Pb | Ca | P |  |  |  |  |
| (Pb_0.00_Ca_1.00_)_5_(PO_4_)_3_OH | 5040 | 5.61 | 0.0000000 | 0.949 | 1.461 | -57.85 | -57.22 | -6311.58 | -6308.02 |
|  | 5760 | 5.86 | 0.0000000 | 0.955 | 1.466 | -56.14 |  | -6301.83 |  |
|  | 7200 | 5.63 | 0.0000000 | 0.962 | 1.465 | -57.68 |  | -6310.64 |  |
| (Pb_0.10_Ca_0.90_)_5_(PO_4_)_3_OH | 5040 | 6.30 | 0.0000194 | 0.255 | 0.241 | -60.09 | -60.30 | -6115.17 | -6116.36 |
|  | 5760 | 6.26 | 0.0000151 | 0.261 | 0.238 | -60.38 |  | -6116.81 |  |
|  | 7200 | 6.25 | 0.0000153 | 0.262 | 0.240 | -60.43 |  | -6117.11 |  |
| (Pb_0.20_Ca_0.80_)_5_(PO_4_)_3_OH | 5040 | 6.56 | 0.0000143 | 0.139 | 0.161 | -62.11 | -62.22 | -5834.44 | -5835.08 |
|  | 5760 | 6.57 | 0.0000142 | 0.114 | 0.161 | -62.38 |  | -5835.98 |  |
|  | 7200 | 6.55 | 0.0000143 | 0.139 | 0.161 | -62.17 |  | -5834.81 |  |
| (Pb_0.30_Ca_0.70_)_5_(PO_4_)_3_OH | 5040 | 6.35 | 0.0000100 | 0.158 | 0.206 | -65.20 | -65.11 | -5587.50 | -5587.02 |
|  | 5760 | 6.38 | 0.0000099 | 0.159 | 0.206 | -65.01 |  | -5586.41 |  |
|  | 7200 | 6.36 | 0.0000099 | 0.159 | 0.206 | -65.13 |  | -5587.14 |  |
| (Pb_0.41_Ca_0.59_)_5_(PO_4_)_3_OH | 5040 | 6.22 | 0.0000119 | 0.144 | 0.174 | -68.54 | -68.36 | -5315.54 | -5314.53 |
|  | 5760 | 6.25 | 0.0000128 | 0.144 | 0.176 | -68.26 |  | -5313.92 |  |
|  | 7200 | 6.25 | 0.0000124 | 0.144 | 0.175 | -68.29 |  | -5314.13 |  |
| (Pb_0.51_Ca_0.49_)_5_(PO_4_)_3_OH | 5040 | 6.31 | 0.0000092 | 0.149 | 0.152 | -70.41 | -70.38 | -5033.97 | -5033.77 |
|  | 5760 | 6.33 | 0.0000101 | 0.138 | 0.158 | -70.20 |  | -5032.78 |  |
|  | 7200 | 6.30 | 0.0000089 | 0.138 | 0.161 | -70.51 |  | -5034.56 |  |
| (Pb_0.61_Ca_0.39_)_5_(PO_4_)_3_OH | 5040 | 6.52 | 0.0000071 | 0.097 | 0.100 | -72.40 | -71.72 | -4808.44 | -4804.55 |
|  | 5760 | 6.56 | 0.0000127 | 0.098 | 0.101 | -71.37 |  | -4802.55 |  |
|  | 7200 | 6.56 | 0.0000124 | 0.097 | 0.102 | -71.39 |  | -4802.65 |  |
| (Pb_0.69_Ca_0.31_)_5_(PO_4_)_3_OH | 5040 | 5.37 | 0.0000881 | 0.238 | 0.445 | -75.56 | -75.48 | -4531.76 | -4531.33 |
|  | 5760 | 5.38 | 0.0000937 | 0.231 | 0.435 | -75.44 |  | -4531.10 |  |
|  | 7200 | 5.37 | 0.0000938 | 0.239 | 0.451 | -75.45 |  | -4531.12 |  |
| (Pb_0.80_Ca_0.20_)_5_(PO_4_)_3_OH | 5040 | 5.18 | 0.0001224 | 0.197 | 0.341 | -78.59 | -78.65 | -4341.05 | -4341.38 |
|  | 5760 | 5.18 | 0.0001240 | 0.196 | 0.328 | -78.62 |  | -4341.23 |  |
|  | 7200 | 5.16 | 0.0001261 | 0.197 | 0.327 | -78.73 |  | -4341.86 |  |
| (Pb_0.89_Ca_0.11_)_5_(PO_4_)_3_OH | 5040 | 5.76 | 0.0000437 | 0.058 | 0.023 | -81.69 | -81.17 | -4065.29 | -4062.31 |
|  | 5760 | 5.78 | 0.0000652 | 0.068 | 0.023 | -80.72 |  | -4059.76 |  |
|  | 7200 | 5.80 | 0.0000508 | 0.066 | 0.023 | -81.09 |  | -4061.87 |  |
| (Pb_1.00_Ca_0.00_)_5_(PO_4_)_3_OH | 5040 | 5.67 | 0.0017452 | 0.000 | 0.007 | -77.42 | -77.51 | -3777.57 | -3778.08 |
|  | 5760 | 5.67 | 0.0015960 | 0.000 | 0.007 | -77.60 |  | -3778.61 |  |
|  | 7200 | 5.66 | 0.0017278 | 0.000 | 0.007 | -77.51 |  | -3778.06 |  |

**Appendix C-2. Supplementary data**

Analytical data and solubility determination of the hydroxypyromorphite–hydroxyapatite solid solution [(Pb_x_Ca_1‒x_)_5_(PO_4_)_3_OH]

(25˚C and an initial pH of 9)

| Sample | Dissolution time (h) | pH | Concentration (mmol/L) | | | log*K*_sp_ | Average log*K*_sp_ | ΔG*_f_^o^* (kJ/mol) | Average ΔG*_f_^o^* (kJ/mol) |
| --- | --- | --- | --- | --- | --- | --- | --- | --- | --- |
|  |  |  | Pb | Ca | P |  |  |  |  |
| (Pb_0.00_Ca_1.00_)_5_(PO_4_)_3_OH | 5040 | 5.84 | 0.0000000 | 0.814 | 1.248 | -54.76 | -56.77 | -6305.45 | -6305.55 |
|  | 5760 | 5.84 | 0.0000000 | 0.818 | 1.244 | -55.04 | -56.77 | -6305.42 |  |
|  | 7200 | 5.83 | 0.0000000 | 0.818 | 1.246 | -59.80 | -56.83 | -6305.78 |  |
| (Pb_0.10_Ca_0.90_)_5_(PO_4_)_3_OH | 5040 | 6.40 | 0.0000125 | 0.215 | 0.163 | -59.55 | -60.34 | -6116.60 | -6116.18 |
|  | 5760 | 6.41 | 0.0000112 | 0.214 | 0.164 | -59.89 | -60.30 | -6116.34 |  |
|  | 7200 | 6.43 | 0.0000115 | 0.215 | 0.164 | -59.75 | -60.16 | -6115.58 |  |
| (Pb_0.20_Ca_0.80_)_5_(PO_4_)_3_OH | 5040 | 6.44 | 0.0000106 | 0.123 | 0.153 | -60.79 | -63.27 | -5841.10 | -5840.71 |
|  | 5760 | 6.45 | 0.0000107 | 0.123 | 0.153 | -61.19 | -63.20 | -5840.68 |  |
|  | 7200 | 6.46 | 0.0000106 | 0.123 | 0.154 | -61.39 | -63.14 | -5840.35 |  |
| (Pb_0.30_Ca_0.70_)_5_(PO_4_)_3_OH | 5040 | 6.31 | 0.0000031 | 0.150 | 0.230 | -60.97 | -66.15 | -5592.96 | -5593.07 |
|  | 5760 | 6.30 | 0.0000033 | 0.150 | 0.230 | -61.31 | -66.18 | -5593.10 |  |
|  | 7200 | 6.30 | 0.0000033 | 0.150 | 0.230 | -62.07 | -66.19 | -5593.14 |  |
| (Pb_0.41_Ca_0.59_)_5_(PO_4_)_3_OH | 5040 | 6.46 | 0.0000065 | 0.111 | 0.164 | -63.47 | -67.91 | -5311.96 | -5312.29 |
|  | 5760 | 6.48 | 0.0000063 | 0.110 | 0.165 | -62.89 | -67.82 | -5311.44 |  |
|  | 7200 | 6.42 | 0.0000064 | 0.111 | 0.165 | -63.80 | -68.18 | -5313.49 |  |
| (Pb_0.51_Ca_0.49_)_5_(PO_4_)_3_OH | 5040 | 6.45 | 0.0000114 | 0.115 | 0.145 | -64.74 | -69.60 | -5029.35 | -5028.98 |
|  | 5760 | 6.44 | 0.0000131 | 0.118 | 0.149 | -64.44 | -69.45 | -5028.48 |  |
|  | 7200 | 6.46 | 0.0000109 | 0.116 | 0.148 | -65.09 | -69.56 | -5029.10 |  |
| (Pb_0.61_Ca_0.39_)_5_(PO_4_)_3_OH | 5040 | 6.44 | 0.0000064 | 0.067 | 0.094 | -65.12 | -73.41 | -4814.18 | -4813.82 |
|  | 5760 | 6.43 | 0.0000070 | 0.067 | 0.098 | -66.05 | -73.29 | -4813.51 |  |
|  | 7200 | 6.40 | 0.0000080 | 0.067 | 0.096 | -66.26 | -73.34 | -4813.78 |  |
| (Pb_0.69_Ca_0.31_)_5_(PO_4_)_3_OH | 5040 | 5.42 | 0.0001378 | 0.215 | 0.441 | -65.43 | -74.61 | -4526.37 | -4526.43 |
|  | 5760 | 5.43 | 0.0001281 | 0.215 | 0.431 | -65.63 | -74.68 | -4526.78 |  |
|  | 7200 | 5.45 | 0.0001254 | 0.215 | 0.432 | -65.67 | -74.57 | -4526.15 |  |
| (Pb_0.80_Ca_0.20_)_5_(PO_4_)_3_OH | 5040 | 5.16 | 0.0001556 | 0.167 | 0.340 | -68.36 | -78.38 | -4339.85 | -4339.74 |
|  | 5760 | 5.15 | 0.0001635 | 0.170 | 0.339 | -66.81 | -78.36 | -4339.73 |  |
|  | 7200 | 5.17 | 0.0001524 | 0.168 | 0.340 | -66.08 | -78.34 | -4339.65 |  |
| (Pb_0.89_Ca_0.11_)_5_(PO_4_)_3_OH | 5040 | 6.35 | 0.0000218 | 0.048 | 0.013 | -58.59 | -79.93 | -4055.24 | -4060.98 |
|  | 5760 | 6.35 | 0.0000122 | 0.015 | 0.011 | -58.96 | -81.50 | -4064.22 |  |
|  | 7200 | 6.29 | 0.0000159 | 0.015 | 0.011 | -59.41 | -81.37 | -4063.49 |  |
| (Pb_1.00_Ca_0.00_)_5_(PO_4_)_3_OH | 5040 | 6.30 | 0.0001906 | 0.000 | 0.006 | -66.06 | -78.17 | -3781.84 | -3781.79 |
|  | 5760 | 6.36 | 0.0001796 | 0.000 | 0.006 | -68.08 | -77.94 | -3780.54 |  |
|  | 7200 | 6.30 | 0.0001751 | 0.000 | 0.006 | -68.01 | -78.37 | -3782.97 |  |
